# People with HIV have higher percentages of circulating CCR5+ CD8+ T cells and lower percentages of CCR5+ regulatory T cells

**DOI:** 10.1038/s41598-022-15646-0

**Published:** 2022-07-06

**Authors:** Louise E. van Eekeren, Vasiliki Matzaraki, Zhenhua Zhang, Lisa van de Wijer, Marc J. T. Blaauw, Marien I. de Jonge, Linos Vandekerckhove, Wim Trypsteen, Leo A. B. Joosten, Mihai G. Netea, Quirijn de Mast, Hans J. P. M. Koenen, Yang Li, André J. A. M. van der Ven

**Affiliations:** 1 Department of General Internal Medicine, Radboud University Medical Center, Nijmegen, The Netherlands; 2 Department of Laboratory Medicine, Radboud University Medical Center, Nijmegen, The Netherlands; 3 Radboudumc Center for Infectious Diseases, Radboud University Medical Center, Nijmegen, The Netherlands; 4 Radboud Institute for Molecular Life Sciences, Radboud University Medical Center, Nijmegen, The Netherlands; 5 Department of Computational Biology for Individualised Medicine, Centre for Individualised Infection Medicine (CiiM) & TWINCORE, Joint Ventures Between the Helmholtz-Centre for Infection Research (HZI) and the Hannover Medical School (MHH), Hannover, Germany; 6 HIV Cure Research Center, Department of Internal Medicine, and Pediatrics, Ghent University & Ghent University Hospital, Ghent, Belgium; 7 Radboud Institute of Health Sciences, Radboud University Medical Center, Nijmegen, The Netherlands

**Keywords:** Chemokines, Infectious diseases, HIV infections, CD8-positive T cells

## Abstract

CCR5 is the main HIV co-receptor. We aimed to (1) compare CCR5 expression on immune cells between people living with HIV (PLHIV) using combination antiretroviral therapy (cART) and HIV-uninfected controls, (2) relate CCR5 expression to viral reservoir size and (3) assess determinants of CCR5 expression. This cross-sectional study included 209 PLHIV and 323 controls. Percentages of CCR5+ cells (%) and CCR5 mean fluorescence intensity assessed by flow cytometry in monocytes and lymphocyte subsets were correlated to host factors, HIV-1 cell-associated (CA)-RNA and CA-DNA, plasma inflammation markers and metabolites. Metabolic pathways were identified. PLHIV displayed higher percentages of CCR5+ monocytes and several CD8+ T cell subsets, but lower percentages of CCR5+ naive CD4+ T cells and regulatory T cells (Tregs). HIV-1 CA-DNA and CA-RNA correlated positively with percentages of CCR5+ lymphocytes. Metabolome analysis revealed three pathways involved in energy metabolism associated with percentage of CCR5+ CD8+ T cells in PLHIV. Our results indicate that CCR5 is differently expressed on various circulating immune cells in PLHIV. Hence, cell-trafficking of CD8+ T cells and Tregs may be altered in PLHIV. Associations between energy pathways and percentage of CCR5+ CD8+ T cells in PLHIV suggest higher energy demand of these cells in PLHIV.

## Introduction

C–C chemokine receptor 5 (CCR5) plays an important role in human immunodeficiency virus (HIV) infection, as it is the predominant co-receptor for viral entry^1^. The homozygous CCR5Δ32 mutation has been shown to prevent CCR5 surface expression and, thereby, impedes infections with CCR5-tropic HIV-1 strains^2^. Furthermore, individuals bearing the heterozygous CCR5Δ32 mutation, which is associated with lower CCR5 expression both in terms of percentage of CCR5+ cells and CCR5 cell surface levels^3,4^, have a beneficial HIV disease course^5–8^. CCR5 expression is also regulated by age, sex, cytomegalovirus infection, medication, and malignancies^9–15^. Furthermore, various cytokines contribute to the upregulation of CCR5 gene expression^16–18^. For instance, pro-inflammatory cytokines may contribute to increased CCR5 expression as activation of T-cells results in DNA demethylation and thereby upregulation of CCR5 expression^19^. In addition, metabolic products may influence and regulate CCR5 expression: modulation of cholesterol and sphingolipids, constituents of the cell membrane, affects CCR5 cell surface expression^13,20^.

Progressively increased CCR5 expression has been reported in untreated PLHIV, possibly as a result of continuous immune activation^4^, a finding confirmed by studies in primates^21^. cART was shown to downregulate the percentage of CCR5+ lymphocytes, which may be due to either less immune activation or generation of low CCR5 expressing naïve cells^22^. Of note, despite the use of cART, virally suppressed PLHIV still display chronic inflammation and metabolic dysregulation^23,24^, which may affect CCR5 expression^25^. A recent study showed that the percentage of CCR5+ cells varies widely between different CD4+ T cell subsets^26^. Although CD8+ T cell subsets may play an important role in controlling the reservoir and in the development of non-AIDS comorbidities, data on CCR5 expression in CD8+ T cell subsets in virally suppressed PLHIV are lacking so far.

Given the critical role of CCR5 in virally suppressed PLHIV, we assessed CCR5 expression on circulating monocytes and lymphocytes in cART treated PLHIV (n = 209) and compared expression levels to HIV-uninfected controls (n = 323). In addition, we studied whether host factors, including circulating inflammatory protein markers, plasma metabolites and CMV serostatus, are associated with CCR5 expression in both cohorts. Finally, associations between CCR5 expression and HIV-related factors and HIV viral reservoir parameters were tested in PLHIV.

## Methods

### Study design and participants

A total of 209 virally suppressed PLHIV (200HIV cohort) were included between December 2015 and February 2017 at the HIV-outpatient clinic of the Radboud University Medical Center (Radboudumc), Nijmegen, the Netherlands, as described before^27^. Inclusion criteria were age $\ge$ 18 years, Western European ethnicity, documented HIV-1 infection, receiving cART > 6 months, and a HIV-RNA viral load $\le$ 200 copies/mL. Exclusion criteria were signs of an acute or opportunistic infection, antibiotics use in the month prior to the study visit, active hepatitis B or C infection, or pregnancy at the time of study visit. PLHIV were compared with a population-based cohort of healthy individuals of Western European descent (300BCG cohort). The 300BCG cohort consists of 323 healthy individuals, which were included in a study to assess the effect of BCG vaccination on immune function^29^. The study participants were recruited between April 2017 and June 2018 in the Radboudumc. For the present study, only data and samples collected before BCG vaccination were used. Both cohorts were embedded in the Human Functional Genomics Project (https://www.humanfunctionalgenomics.org).

This study was performed in accordance with the principles of the Declaration of Helsinki. Approval was granted by the Medical Research Ethics Committee Oost-Nederland (200HIV cohort: ref. 42561.091.122, and 300BCG cohort: ref. 58553.091.16). Written informed consent was obtained from all study participants.

Demographic data from all study participants were collected by questionnaires while clinical information was extracted from medical files in the electronic hospital information system and the ‘Stichting HIV Monitoring’ registry (Amsterdam, the Netherlands). All data were recorded in the Castor Electronic Data Capture program (Castor EDC, CIWIT B.V., Amsterdam, the Netherlands).

### Cell processing

For both PLHIV and controls, flow cytometry analysis was performed approximately 1–4 h after blood was drawn using the identical antibody panel, and flow cytometry measurements and pre-processing stages were conducted by the same group of trained laboratory technicians.

Venous blood was collected between 8 and 11 am in sterile 10 ml EDTA tubes. After centrifugation, plasma was stored at − 80 °C until follow-up analysis. Cell counts were determined by a Sysmex XN-450 automated hematology analyzer (Sysmex Corporation, Kobe, Japan) and used to calculate the absolute numbers of CD45 + white blood cell (WBC) counts as measured by flow cytometry.

For the lysis of erythrocytes, 1.5 ml EDTA-anticoagulated blood was incubated for 10 min in lysis buffer with 3.0 M NH_4_Cl, 0.2 M KHCO_3_ and 2 mM Na_4_EDTA. Next, cells were diluted with 25 ml phosphate-buffered saline 1x (PBS, Braun, Melsungen, Germany) and centrifuged at room temperature at 452 × g for 5 min. Cells were again washed, resuspended in 300 μl of PBS enriched with 0.2% bovine serum albumin (BSA, Sigma-Aldrich, Zwijndrecht, Netherlands), and then stained for flow cytometry. The fluorochrome conjugates and clone identity of the antibodies are shown in Supplementary Table S1. Details of the staining procedure were described previously^28^.

### Flow cytometry

Flowcytometry data were acquired with a 10-color Navios flow cytometer (Beckman Coulter), and the Kaluza Flow Cytometry software (Beckman Coulter, version 2.1). Extensive immunophenotyping was done in all study participants in the same way and as previously described^28,29^. For the present study, our analyses were adjusted to allow quantification of CCR5 expression. We analysed CCR5 expression on monocytes and lymphocytes, including CD8+ T cells and five different CD8+ T cell subsets, CD4+ T cells and seven different CD4+ T cell subsets (Supplementary Fig. S1).

Sequential manual gating was performed to identify different cell subsets (Supplementary Fig. S1). Leukocytes were identified by first gating on live and single cells and subsequently on CD45 + cells. Within the CD45 + gated cells, lymphocytes and monocytes were identified by granularity (side scatter) and size (forward scatter). Lymphocytes were further classified into CD4+ (CD8-) T cells and (CD4-) CD8+ T cells. The cell surface markers CD45RA and CCR7 were used to further classify CD4+ T cells and CD8+ T cells as being naïve (CD45RA + CCR7 +), central memory (CM, CD45RA-CCR7 +), effector memory cells (EM, CD45RA-CCR7-), effector memory cells expressing CD45RA (TEMRA, CD45RA + CCR7-) and the total pool of effector memory cells (TEM, CD45RA−/+CCR7−)^30,31^. Furthermore, CD4+ naive regulatory (nTreg, CD45RA+ CD25+) and CD4+ memory regulatory (mTreg, CD45RA-CD45++) T cell subsets were identified within the subset of CD4+ CD8- T cells.

Subsequently, gates for CCR5 were set using granulocytes as an internal negative control. The regions that identified CCR5- cell populations in granulocytes were applied to other cell types to distinguish between CCR5- and CCR5+ cell populations in a standardized and reproducible manner. We also applied our protocol to three fluorescence minus one (FMO) controls. The percentage of CCR5+ cells (%) and CCR5 geometric mean fluorescence intensity (MFI) representing cell surface density were determined on all identified cell types (Supplementary Fig. S1).

### Proteomic profiling of the circulating plasma inflammatory markers

EDTA plasma samples from PLHIV and healthy controls were subjected to proteome analysis using the commercially available Olink® Inflammation Panel that includes 92 inflammatory proteins (Olink Bioscience AB, Uppsala, Sweden). Detected proteins are measured on a log2 scale as normalized protein expression values (NPX). When the target protein was detected in > 70% of the samples in both PLHIV and controls, it was included in our follow-up analysis, resulting in sixty-nine out of 92 proteins. In addition, Olink proteomics performed a quality control per sample: samples that deviated less than 0.3 normalized protein expression units (NPX) from the plate median passed the quality control. Eight samples of the PLHIV and three samples of the controls did not pass this quality control and were excluded from further analysis.

RANTES, a CCR5 ligand, was determined in PLHIV only by ELISA (R and D systems, Minneapolis, USA) in citrated plasma samples according to the manufacturer’s instructions^32^.

### Viral reservoir

Viral reservoir quantification was performed in all participating PLHIV as previously described^27^. In short, HIV-1 cell-associated (CA) RNA and CA-DNA were measured by droplet digital PCR in isolated CD4+ T cells. CA-DNA and CA-RNA measurements were normalized using reference genes (*RPP30* for CA-DNA, and *B2M, ACTB,* and *GADPH* for CA-RNA) and expressed per million PBMCs. Also, the ratio between CA-RNA and CA-DNA was calculated.

### Metabolomics

EDTA plasma samples of PLHIV and healthy controls were frozen and stored before metabolite identification. Participants were not restricted to their food- and/or drinks consumption before blood samples were drawn. Untargeted metabolomics was performed by flow injection electrospray – time-of-flight mass spectrometry to identify metabolites in plasma samples of participants in collaboration with General Metabolics, LLC, and executed at General Metabolics (Boston, Massachusetts, United States), according to the methodology described previously^33^. Metabolites were identified based on the mass-to-charge ratio (ion m/z)^33^. Means of duplicate peak intensity values were calculated and normalization of the samples from PLHIV and controls was performed using a moving median and quantile normalization respectively. We performed principal component analysis (PCA) to evaluate the possibility of batch effects within each cohort and observed no clear batch effect.

The pathways analysis using the summary statistics of the correlation analysis between metabolic compounds and CCR5 variables was performed independently for each CCR5 variable (see Quantification and Statistical Analysis for details). We selected the MS Peaks to Pathway module, which predicts pathway activity from raw mass spectra (MS) using the Mummichog algorithm from MetaboAnalyst 5.0^34^. In this module, MS peaks are putatively annotated and mapped to the human Kyoto Encyclopedia of Genes and Genomes (KEGG) database^35^. By this approach, different MS peaks may be mapped to the same KEGG ID. The following settings were applied: negative ion mode, mass tolerance of 5.0 ppm and the *p* value cut-off was based on the top 10% MS peaks.

### Quantification and Statistical Analysis

Statistical analyses were performed in R version 3.6.0^36^. Continuous variables were summarized as medians and compared between PLHIV and healthy controls using a Mann–Whitney U test. Binary variables were summarized as percentages and compared between PLHIV and controls using a chi-squared test. R-package RNOmni was used to perform inverse rank-based transformation (IRT) of expression data, which is recommended for continuous traits with non-normally distributed residuals^37^.

#### Linear regression analysis

We first compared IRT-transformed CCR5 expression data between PLHIV and healthy controls using a linear regression analysis. Since the patient and control groups differed in demographic composition, we used a linear model with age, sex, CMV serostatus (CMV IgG positive/negative) and current smoking status (current smoker yes/no) as covariables:

$$IRT\,CCR5\,variable \sim Cohort + Age + Sex + CMV\,serostatus + Smoking\,status$$

Secondly, we assessed the associations between HIV-specific characteristics and CCR5 expression using a linear regression model. Variables that significantly correlated to CCR5 expression were added to the linear model as explanatory variables:

$$IRT\,CCR5\,variable \sim HIV\,specific\,characteristics$$

#### Spearman’s correlation analysis

We performed correlation analyses in PLHIV and controls using Spearman’s correlation to evaluate associations between CCR5 expression and (1) host factors (including the viral reservoir and HIV-specific characteristics in PLHIV), (2) circulating protein inflammation markers, and (3) metabolic compounds. To control for multiple testing, *p* values were corrected using the false discovery rate (FDR) test unless indicated otherwise. Specifically, CCR5 expression was correlated with the following variables as described below:

1. *Host factors.* The CCR5 expression variables were correlated to host factors, including age, sex, BMI, CMV serostatus, and current smoking status in both PLHIV and healthy controls. In addition, correlation between CCR5 expression and HIV-specific factors, such as way of transmission, cART duration, CD4 nadir, latest CD4 nadir, latest CD4:CD8 ratio, HIV-RNA zenith and past HBV infection was performed in PLHIV. No covariables were added in these analyses. Also, CCR5 expression was correlated to HIV-1 cell associated (CA) RNA, CA-DNA and the HIV-1 CA-RNA / CA-DNA ratio, as measurements of the viral reservoir size, using a partial correlation test with age, sex, and CMV serostatus as covariables.
2. *Circulating inflammatory protein markers.* Circulating inflammatory proteins were correlated to CCR5 expression using age and sex as covariables.
3. *Metabolic compounds.* Metabolic compounds were correlated to CCR5 expression while adjusting for age, sex, and CMV serostatus. Unadjusted *p* values obtained from the correlation analysis were extracted and used for subsequent pathway analysis.

## Results

### General characteristics of the study populations

The baseline characteristics of 209 PLHIV and 323 controls, including HIV-specific characteristics of PLHIV, are summarized in Table 1. PLHIV were older (median age = 52 years), 91% of PLHIV were males, and were more often seropositive for cytomegalovirus (CMV). The majority of PLHIV (157/209, 75%) were men having sex with men and contracted HIV by homosexual contact. Other routes of transmission included heterosexual contact (39/209), intravenous drug use (IDU, 3/209), needle stick injury (1/209), contaminated blood products (1/209) and for 8/209 participants the way of transmission was unknown. In addition, PLHIV showed the following parameters: median of CD4 nadir 250 10^6 cells/L and IQR: 230, median of latest CD4 660 10^6 cells/L and IQR: 330, and median of zenith HIV-RNA 100.000 copies/ml and IQR: 345.591. PLHIV used cART for a median duration of 6.61 years (IQR 7.70), with 67% (139/209) using integrase inhibitor, 30% (63/209) non-nucleoside analogue and 15% (32/209) a protease inhibitor. Almost all participants (203/209) were having an HIV-RNA viral load beneath the detection limit (20 copies/mL until, and 40 copies/mL after March 14, 2016). Viral reservoirs in PLHIV were measured as cell-associated HIV-1 RNA, CA-HIV-1 DNA and the RNA:DNA ratio. PLHIV had CA-HIV-1 RNA of 2.192 [1.875–2.485], CA-HIV-1 DNA, 3.184 [2.776–3.467] (both expressed as median log10 copies/10^6^ CD4 cells, and a RNA:DNA median ratio of 0.118276 [0.067587–0.186814]. Regarding cell counts, PLHIV had significantly less naïve CD4+ and CD8+ T cells, and less CD4+ nTregs and mTregs, while they had more central memory (CM) cells (CD4+ and CD8 +), CD8+ effector memory (EM) cells (including CD8+ EM, TEMRA, and TEM cells), and CD8+ T cells, compared to controls (Table 1).

**Table 1:** Baseline characteristics in PLHIV and controls.

	PLHIV, n = 209	Healthy controls, n = 323	*p* value	95% confidence interval
Age (years)	52.47 (13.86)	23.30 (4.40)	< 2 × 10^–16^	26.44–29.54
Sex, % males (males/total)	90.91 (190/209)	43.34 (140/323)	< 2 × 10^–16^	NA
BMI (kg/m^2)^	24.15 (4.08)	22.21 (2.86)	4.053 × 10^–12^	1.31–2.30
CMV status, % positive IgG (positive IgG/total)	94.23 (196/208)	24.05 (76/316)	< 2 × 10^–16^	NA
Smoking, % smokers (active smokers/total)	28.88 (60/208)	6.19 (20/323)	2.573 × 10^–12^	NA
WBC (10^9^/L)	6.40 (2.80)	6.10 (2.35)	0.1889	− 0.10 to 0.60
Monocytes (10^9^/L)	0.46 (0.36)	0.42 (0.24)	0.1111	− 0.01 to 0.07
Lymphocytes (10^9^/L)	3.00 (1.54)	2.25 (1.36)	1.192 × 10^–15^	0.58 to 0.93
CD4+ T cells (10^9^/L)	1.17 (0.81)	1.08 (0.71)	0.6599	− 0.11 to 0.07
Naïve CD4+ T cells (10^9^/L)	0.36 (0.45)	0.49 (0.41)	4.338 × 10^–05^	− 0.15 to − 0.06
CM CD4+ T cells (10^9^/L)	0.24 (0.16)	0.20 (0.15)	0.001372	0.01–0.05
EM CD4+ T cells (10^9^/L)	0.36 (0.21)	0.35 (0.21)	0.4992	− 0.02 to 0.04
TEMRA CD4+ T cells (10^9^/L)	0.05 (0.06)	0.05 (0.04)	0.5535	− 0.00 to 0.01
TEM CD4+ T cells (10^9^/L)	0.43 (0.25)	0.41 (0.25)	0.3546	− 0.02 to 0.05
nTreg CD4+ T cells (10^9^/L)	0.02 (0.02)	0.02 (0.02)	0.0002	− 0.01 to 0.00
mTreg CD4+ T cells (10^9^/L)	0.03 (0.02)	0.04 (0.02)	1.696 × 10^–10^	− 0.01 to − 0.01
CD8+ T cells (10^9^/L)	1.06 (0.75)	0.54 (0.37)	< 2 × 10^–16^	0.47–0.61
Naïve CD8+ T cells (10^9^/L)	0.20 (0.20)	0.24 (0.20)	0.0002	− 0.07 to − 0.02
CM CD8+ T cells (10^9^/L)	0.04 (0.06)	0.02 (0.02)	< 2 × 10^–16^	0.02–0.03
EM CD8+ T cells (10^9^/L)	0.35 (0.24)	0.16 (0.12)	< 2 × 10^–16^	0.15–0.21
TEMRA CD8+ T cells (10^9^/L)	0.45 (0.42)	0.06 (0.08)	< 2 × 10^–16^	0.31–0.40
TEM CD8+ T cells (10^9^/L)	0.79 (0.67)	0.24 (0.18)	< 2 × 10^–16^	0.48–0.59

To test for significant differences between the two groups, Mann–Whitney U test was used for continuous variables and chi-square test for binominal categorical variables. Data were summarized as medians and IQR. PLHIV = people living with HIV, BMI = body mass index, CMV = cytomegalovirus, WBC = white blood cell count, CM T cells = central memory T cells, EM T cells = effector memory T cells, TEMRA cells = T effector memory cells expressing CD45RA, nTreg = naïve regulatory T cells, mTreg = memory regulatory T cells. Differences in cell counts of nTreg CD4+ T cells, mTreg CD4+ T cells and naïve CD8+ T cells were small. For PLHIV and HC respectively, the counts of these cell types with four decimal places were: 0.0187 (0.0225) 10^9^/L and 0.0239 (0.0218) 10^9^/L nTreg CD4+ T cells, 0.0296 (0.0248) 10^9^/L, and 0.0396 (0.0235) 10^9^/L mTreg CD4+ T cells, and 0.1953 (0.2046) 10^9^/L and 0.2431 (0.2015) 10^9^/L naïve CD8+ T cells. For HIV-RNA load, detection limits were 20 copies/mL until, and 40 copies/mL after March 14, 2016.

### Increased percentages of CCR5+ CD8+ T cells and monocytes were identified in PLHIV compared to controls

**Figure 1:**
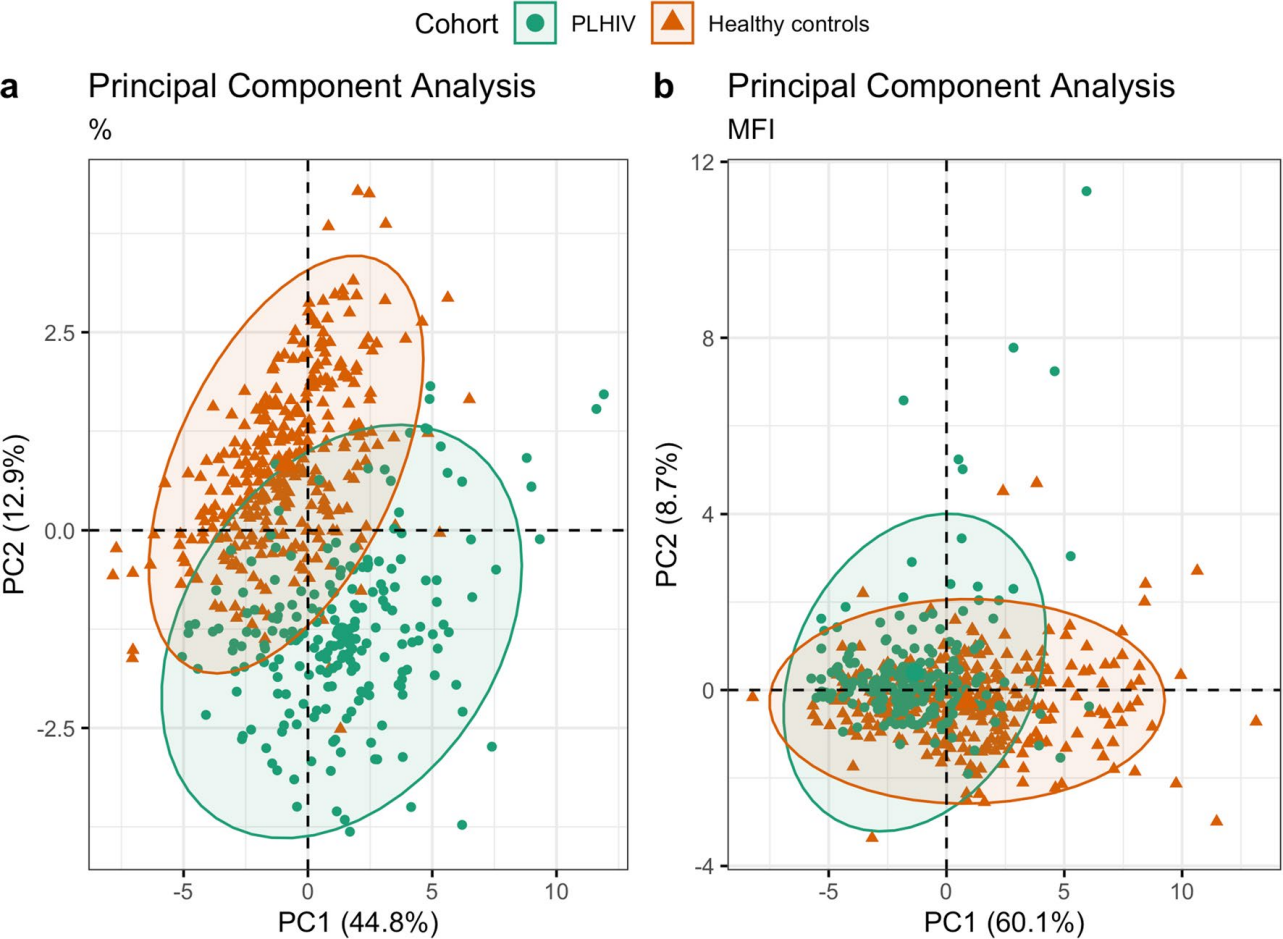
Principal components analysis (PCA) of CCR5 expression levels, separately performed for (**a**) percentage of CCR5+ cells and (**b**) level of CCR5 expression on positive-gated cells (MFI), reveals distinct patterns of CCR5 expression in PLHIV versus controls. Each datapoint represents an individual of either the controls (orange) or PLHIV (green). Parameters included in the PCA are CCR5 expression (percentage for (**a**) and MFI for (**b**) on the following cell types: CD45 + cells, monocytes, lymphocytes, CD4+ T cells, including naïve, CM, EM, TEMRA, TEM, nTreg, and mTreg CD4+ cells, and CD8+ T cells, including naïve, CM, EM, TEMRA, and TEM CD8+ T cells. The x- and y-axes of plots a and b represent the percentage of variance explained by principal component 1 and 2, respectively. The contribution (expressed as percentage) of each parameter to principal components 1 and 2 is given in Supplementary Table S2.

**Figure 2:**
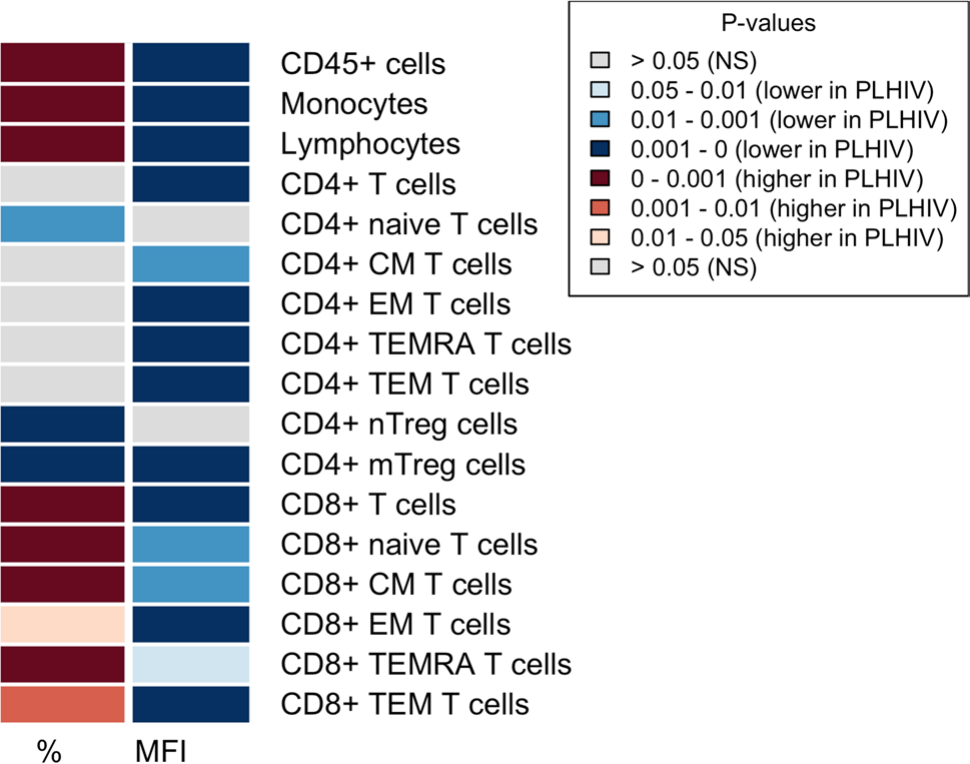
CCR5 expression in circulating immune cells in PLHIV versus controls. Multiple linear regression analysis was used to evaluate the effect of cohort (PLHIV vs. controls) on CCR5 expression levels (percentage (%) of CCR5+ cells or CCR5 mean fluorescence intensity (MFI)) on cell types listed in the rows, when adjusting for age, sex, CMV-serostatus, and smoking. The columns show the differences in the percentage of CCR5+ cells (left column) and CCR5 MFI (right column) between PLHIV and controls. Significance (FDR corrected) of the difference in CCR5 expression between PLHIV and controls is presented by the colours. Blue colour refers to lower CCR5 expression in PLHIV, while red refers to higher expression in PLHIV compared to controls.

CCR5 expression on various circulating immune cells was compared between PLHIV and controls. We first performed principal component analysis (PCA) to assess the overall variance among cell subtypes on CCR5 MFI and percentage of CCR5+ cells. The PCA plots showed clear differences in variance in CCR5 expression between PLHIV and controls. However, differences were more distinct for percentage of CCR5+ cell subsets (Fig. 1a) than for MFI (Fig. 1b). Indeed, significant differences were observed in CCR5 expression of various circulating immune cells between PLHIV and controls (Table 2, Supplementary Fig. S2 and S3), which remain after adjusting for age, sex, CMV serostatus and smoking (*P* < 0.05, Fig. 2). Looking specifically at CCR5 positivity in the various subsets, we found that the percentage of CCR5+ CD45 + cells, monocytes, lymphocytes, and CD8+ T cells, including naïve, CM, EM, TEMRA, and the total population of CD8+ EM (TEM) T cells was higher in PLHIV compared to controls, while it was lower in naïve CD4 cells and naïve and memory Tregs (Fig. 2, left column). Higher percentages of CCR5+ cells between PLHIV and controls were especially pronounced in CCR5+ CD8+ naïve T cells and CD8+ CM T cells (Table 2, Supplementary Fig. SS2). In contrast, the MFI of CCR5+ cells was lower in most cell types from PLHIV compared to controls (Fig. 2, right column). The largest relative difference in MFI was found in the general population of CD8+ T cells, and the total population of CD8+ EM T cells (TEM cells) (Table 2, Supplementary Fig. S3). Because sex differed between PLHIV and controls, we performed an additional analysis in males only to compare CCR5 expression between PLHIV and controls (Supplementary Fig. S4). This analysis weakened the differences in MFI but exaggerated the differences for percentages of CCR5+ CD8+ T cells, further highlighting the altered CCR5+ CD8+ T cells subsets in PLHIV.

**Table 2:** CCR5 expression levels (%CCR5+ cells and MFI) in PLHIV and controls.

			PLHIV, n = 209	Controls, n = 323	*p* value	95% confidence interval
gMFI		CD45 + (gMFI)	1.72 (0.40)	2.03 (0.65)	1.38e−14	− 0.36 to − 0.22
		Monocytes (gMFI)	1.47 (0.32)	1.65 (0.57)	3.67e−06	− 0.21 to − 0.09
		Lymphocytes (gMFI)	1.87 (0.47)	2.32 (0.72)	< 2.20e−16	− 0.54 to − 0.37
	CD4+	CD4+ (gMFI)	1.59 (0.38)	1.86 (0.585)	4.47e−12	− 0.32 to − 0.18
		CD4+ naive (gMFI)	1.21 (0.85)	1.25 (0.59)	0.96	− 0.07 to 0.08
		CD4+ CM (gMFI)	1.28 (0.31)	1.45 (0.58)	2.61e−08	− 0.23 to − 0.11
		CD4+ EM (gMFI)	1.61 (0.40)	1.91 (0.595)	2.40e−15	− 0.35 to − 0.21
		CD4+ TEMRA (gMFI)	1.71 (0.57)	2.19 (0.77)	< 2.20e−16	− 0.51 to − 0.33
		CD4+ TEM	1.61 (0.38)	1.94 (0.595)	< 2.20e−16	− 0.37 to − 0.23
		CD4+ nTreg (gMFI)	1.71 (1.09)	1.69 (0.835)	0.22	− 0.04 to 0.20
		CD4+ mTreg (gMFI)	1.72 (0.45)	1.96 (0.64)	4.62e−08	− 0.27 to − 0.13
	CD8+	CD8+ (gMFI)	2.06 (0.59)	2.71 (1.16)	< 2.20e−16	− 0.80 to − 0.55
		CD8+ naive (gMFI)	1.67 (0.57)	1.97 (1.575)	4.23e−05	− 0.42 to − 0.14
		CD8+ CM (gMFI)	1.71 (0.54)	1.8 (0.86)	0.28	− 0.14 to 0.04
		CD8+ EM (gMFI)	2.33 (0.80)	2.9 (1.29)	7.56e−15	− 0.69 to − 0.42
		CD8+ TEMRA (gMFI)	1.83 (0.56)	1.95 (0.745)	< 0.01	− 0.24 to − 0.06
		CD8+ TEM (gMFI)	2.08 (0.63)	2.73 (1.175)	< 2.20e−16	− 0.79 to − 0.53
%		CD45 + (%)	24.86 (9.55)	13.22 (7.615)	< 2.20e−16	9.34–11.77
		Monocytes (%)	72.9 (12.28)	64.28 (15.675)	1.37e−13	5.86–9.85
		Lymphocytes (%)	26.09 (12.16)	17.94 (9.185)	< 2.20e−16	6.37–9.24
	CD4+	CD4+ (%)	13.20 (10.36)	12.13 (7.07)	< 0.01	0.51–2.69
		CD4+ naive (%)	1.12 (0.73)	1.47 (1.265)	7.27e−06	− 0.44 to − 0.17
		CD4+ CM (%)	3.91 (2.40)	4.21 (2.665)	0.27	− 0.53 to 0.15
		CD4+ EM (%)	31.80 (17.63)	31.26 (14.82)	0.13	− 0.47 to 3.70
		CD4+ TEMRA (%)	31.01 (23.44)	25.48 (17.725)	8.52e−08	4.67–10.09
		CD4+ TEM	32.50 (18.46)	30.4 (14.22)	0.01	0.78–4.91
		CD4+ nTreg (%)	2.18 (3.01)	7.73 (7.05)	< 2.20e−16	− 5.63 to − 4.23
		CD4+ mTreg (%)	24.5 (15.37)	32.86 (17.84)	2.54e−10	− 9.67 to − 5.20
	CD8+	CD8+ (%)	42.32 (17.31)	21.98 (14.625)	< 2.20e−16	16.58–21.00
		CD8+ naive (%)	2.81 (3.55)	0.26 (0.34)	< 2.20e−16	2.02–2.70
		CD8+ CM (%)	21.35 (15.10)	5.17 (6.01)	< 2.20e−16	13.56–16.65
		CD8+ EM (%)	64.49 (22.48)	61.98 (18.255)	0.12	− 0.58 to 4.60
		CD8+ TEMRA (%)	49.82 (23.60)	24.47 (20.97)	< 2.20e−16	20.72–26.66
		CD8+ TEM (%)	55.83 (19.58)	52.41 (19.98)	< 0.01	1.19–6.36

The unadjusted *p* values are derived from comparisons between the groups by Mann–Whitney U test. Data were summarized as medians and IQR. PLHIV = people living with HIV, CM T cells = central memory T cells, EM T cells = effector memory T cells, TEMRA cells = T effector memory cells expressing CD45RA, nTreg = naïve regulatory T cells, mTreg = memory regulatory T cells.

### CCR5 expression in relation to viral reservoir parameters

**Figure 3:**
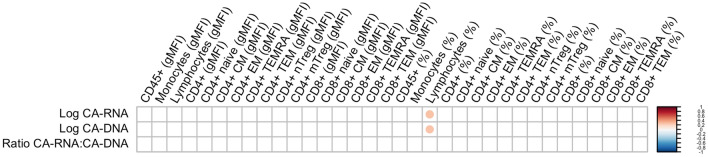
Spearman’s rank correlation plot of viral reservoir parameters with CCR5 expression (MFI and %) on various immune cell subsets. Correlation coefficients were adjusted for age, sex and CMV-serostatus. The size of the circles and the depth of the colour represent the strength of the correlation, while the direction is indicated by colours: red for a positive correlation and blue for a negative correlation. A blank square represents the absence of a significant correlation, while a coloured square represents a significant correlation (FDR-corrected *p* value < 0.05).

We next assessed whether cell associated (CA) HIV-1 DNA and CA-HIV-1 RNA, and the ratio between CA HIV-1 RNA and CA HIV-1 DNA, as measurements of the viral reservoir, correlate to CCR5 expression in PLHIV (Fig. 3). We observed a positive significant correlation between the percentage of CCR5+ cells of the general lymphocyte subset with CA HIV-1 RNA (rho = 0.29, *P* = 0.007) and CA HIV-1 DNA (rho = 0.28, *P* = 0.007).

### Host factors correlate with the percentage of CCR5+ cells in both controls and PLHIV

**Figure 4:**
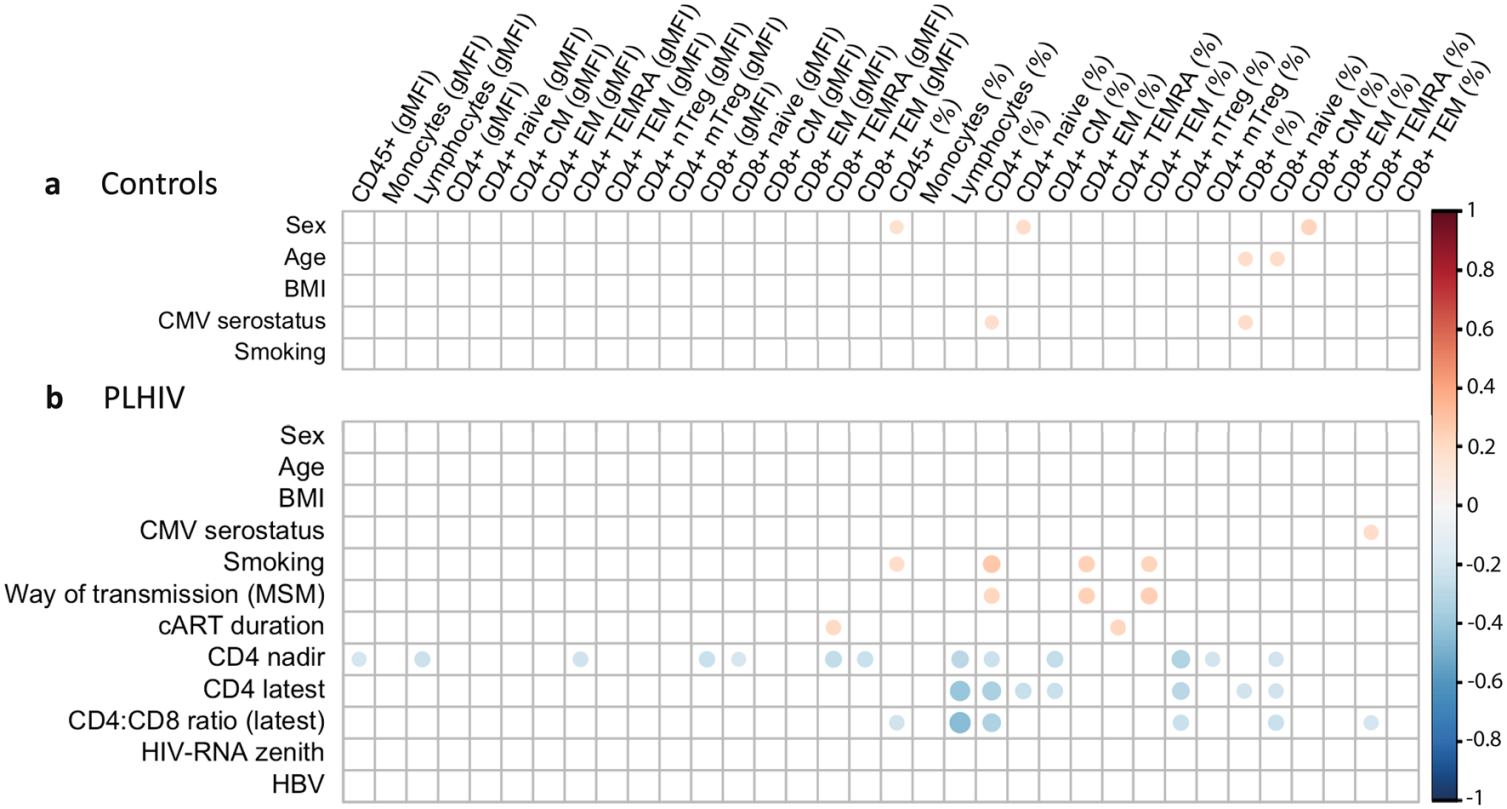
Spearman’s rank correlation plots of host factors with CCR5 expression (MFI and %) of immune cell subsets in (**a**) controls and (**b**) PLHIV. The size of the circles and the depth of the colour represent the strength of the correlation, while the direction is indicated by colours: red for a positive correlation and blue for a negative correlation. A blank square represents the absence of a significant correlation, while a coloured square represents a significant correlation (FDR-corrected *p* value < 0.05).

We next determined whether host factors are correlated to CCR5 expression. In controls, age, male sex, and CMV serostatus showed significant positive correlations with the percentage of different CCR5+ CD4+ and CD8+ T cell subsets (adjusted *P* < 0.05) (Fig. 4), unlike in PLHIV in whom HIV-specific characteristics were significant: cART duration was positively correlated with percentages of CCR5+ TEMRA cells (both CD4+ and CD8 +) while CD4 cell count, including nadir, latest, and the CD4/CD8 ratio, correlated negatively to percentages of various CCR5+ CD4+ and CCR5+ CD8+ T cell subsets. We also assessed the correlation between CCR5 expression (%CCR5+ cells and MFI) and the different cART regimens and found no significant association after FDR-correction (data not shown).

Given that different HIV-specific factors, such as CD4 nadir and CD4 latest, may relate to each other, we performed a multiple linear regression analysis to assess which host factors remain significantly related to percentages of CCR5+ cell subsets when adjusting for HIV-specific variables. All HIV-specific variables that were significantly correlated to one or more variables, were added to this analysis. As presented in Supplementary Fig. S5, HIV transmission risk behaviour and low CD4 nadir showed significant associations with the percentage of CCR5+ cells and CCR5 MFI of several cell subsets.

### Circulating inflammatory proteins weakly correlate to CCR5 expression in both controls and PLHIV

Targeted proteome analysis was performed in PLHIV and controls. To investigate whether inflammatory markers were correlated to CCR5 expression on immune cell subtypes, we performed Spearman’s correlation analysis in PLHIV and controls. Overall, we observed weak correlations in PLHIV and controls that did not remain significant after FDR-correction (Supplementary Fig. S6). The most significant correlation in PLHIV was observed for the CCR5 ligand CCL4 with the percentage of CCR5+ CD8+ T cells (rho = − 0.28, *P* < 0.01), and for controls between TNF-related activation-induced cytokine (TRANCE) and the percentage of CCR5+ CD8+ total EM cells (rho = -0.21, *P* < 0.01). Similar to CCL4, RANTES correlated negatively with the percentage of various CCR5+ immune cell types (CCR5+ CD45 + T cells, CD8+ T cells, and CD8+ total EM cells) in PLHIV.

### Different metabolic pathways relate to CCR5 expression in PLHIV and controls

**Figure 5:**
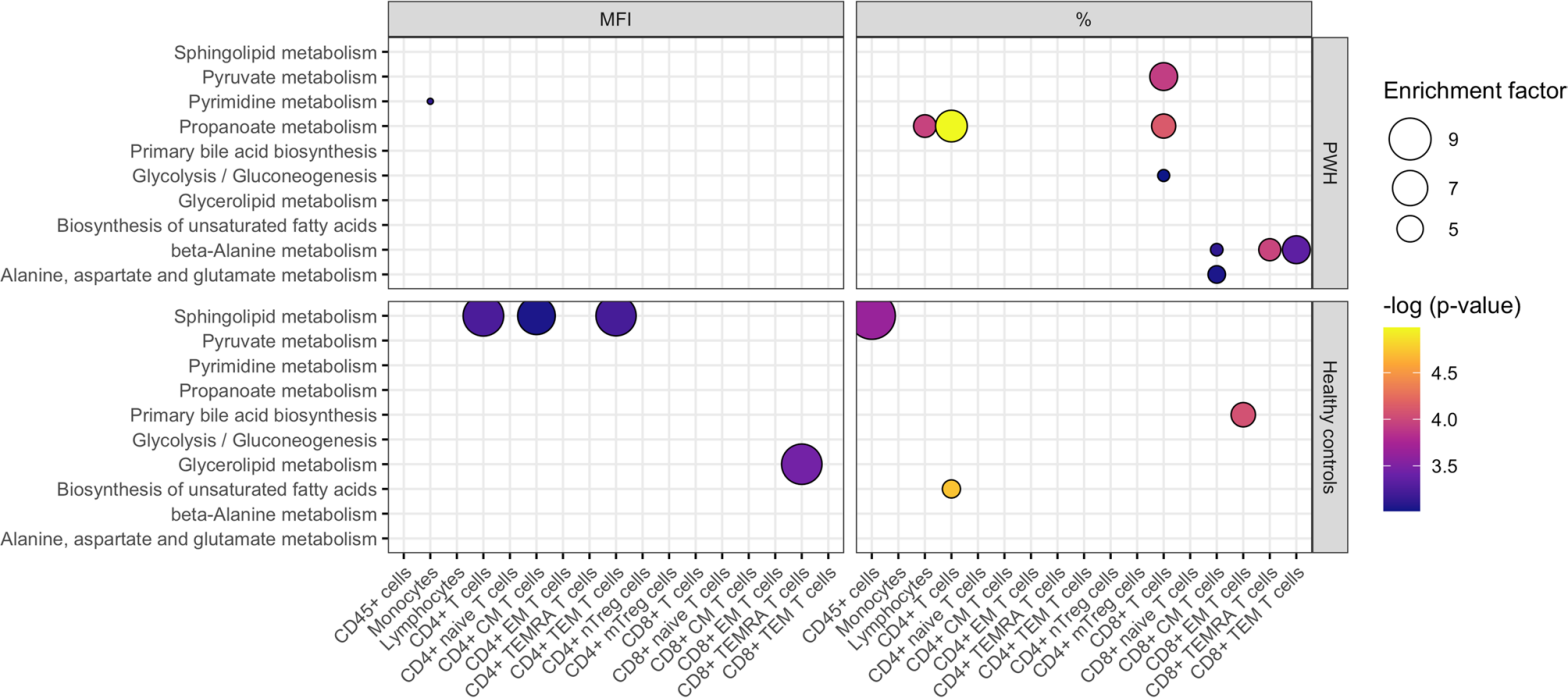
Metabolic pathways associated to CCR5 expression (MFI left panels, % right panels) from various immune cell subsets. CCR5 expression was measured as MFI (left panels) and percentage of CCR5+ cells (%, right panels)). The upper panels show results for PLHIV, whereas the lower panels show the results from controls. The colour of the circles depicts the significance of the association between pathways and CCR5 expression. The size of the circles depicts the enrichment factor, which refers to the ratio between the number of significant pathway hits and the expected number of compound hits within the pathway (no cut-off value). Unadjusted *p* values are presented, with a cut-off < 0.001 being used for the selection of the pathways.

A total of 1659 and 1607 metabolic compounds were identified in samples of PLHIV (n = 205) and controls (n = 325) respectively. To investigate the metabolic pathways that are associated with CCR5 expression in PLHIV and controls in different immune cells, we performed metabolic pathway activity predictions using the Mummichog approach^38^. First, metabolic pathways involved in percentage of CCR5+ cells were evaluated. Five pathways were found for PLHIV (Fig. 5), including three pathways that are relevant to energy metabolism (glycolysis/gluconeogenesis, pyruvate, and propanoate). Three other pathways were found for controls (Fig. 5). Regarding CCR5 MFI, we found striking differences between PLHIV and controls: sphingolipid metabolism was highlighted in controls, but not in PLHIV (Fig. 5, Supplementary Table S3). To further evaluate the relationship between metabolites involved in the identified pathways and CCR5 expression, we assessed correlations between these specific metabolic compounds (IonMz values) and CCR5 expression (Supplementary Fig. S7–S10). For the percentage of CCR5+ cells, we found positive correlations between metabolic compounds involved in propanoate (Supplementary Fig. S7), pyruvate (Supplementary Fig. S8), and beta-alanine metabolism (Supplementary Fig. S9) and percentages of CCR5+ CD8+ T cells in PLHIV. For CCR5 MFI, metabolic compounds involved in the sphingolipid pathway were negatively correlated to CCR5 MFI on several CD4+ cell subsets in controls (Supplementary Fig. S10).

## Discussion

We aimed to compare the expression of CCR5 on circulating immune cells between PLHIV on suppressive cART and HIV-uninfected controls and found that PLHIV have increased percentages of CCR5+ CD8+ T cells, in contrast to decreased percentages of CCR5+ Tregs. HIV-specific factors, as well as metabolic compounds, seem to modify CCR5 expression, unlike inflammation.

First, we compared CCR5 expression between PLHIV and controls. We found that leucocytes, monocytes, and lymphocytes express higher percentages of CCR5 in PLHIV compared to controls. This difference was pronounced in CD8+ T cells, and more specifically in naïve, CM and TEMRA CD8+ T cells. CCR5 expression in the total population of CD4+ T cells did not differ between PLHIV and controls, confirming a pattern suggested by others^22^. However, a further characterization showed lower percentages of CCR5+ naïve CD4 T cells and Tregs. CCR5 signaling mediates the migration of Tregs to sites of inflammation^39^. Hence, reduced CCR5 expression on Tregs can impair homing of Tregs and may weaken their role in controlling tissue inflammation^39^. Absence of CCR5 expression on naive CD4 cells has been suggested as mechanism of resistance against direct infection of these cells^40^. Our findings indicate that CCR5 is present on CD4+ naïve T cells, although at low levels. Contrasting observations have previously been made for CD8+ T cells; some report that cART normalizes CCR5+ CD8+ T cell percentages, though this takes longer than for CCR5+ CD4+ T cells^22^, other studies notice a trend towards higher CCR5+ CD8+ T cell percentages^41^. Our results show that CCR5 expression is not only upregulated in the total CD8+ T cell population, but also in all measured CD8+ T cell subsets. Literature on alterations in CCR5 expression on monocytes in PLHIV is scarcer and more inconclusive. Some report that percentages of CCR5+ monocytes are undetectable to low in both PLHIV and controls^41^, while others find higher levels of CCR5 expression, depending on differentiation and the antibody used for immunophenotyping^42,43^. Our results indicate that 64–72% of monocytes express CCR5. We noticed for several cell types, including Tregs and CD8+ T cell subsets, that cell counts and percentages of CCR5+ cells showed the same trend in the comparison between PLHIV and controls. For example, PLHIV had higher cell counts of CD8+ EM T cells compared to controls, as well as increased percentage of CCR5+ CD8+ EM T cells. We speculate that this similar trend may be due to difference in homing or proliferation^44^.

In contrast to percentage of CCR5 expression, the CCR5 density (MFI) was lower on both monocytes and lymphocytes in PLHIV, including several CD4+ and CD8+ T cell subsets. These differences in MFI were rather limited and the clinical impact may be less clear than alterations in percentages of CCR5+ cells. Previous studies reported that CCR5 MFI on CD4+ and CD8+ T cells is comparable between PLHIV and controls^45,46^.

The CCR5 protein plays an important role in differentiation and activation of CD8+ T cells as has been shown in SIV infected macaques, whereby blocking of CCR5 reduced inflammation, but at the same time also impaired virus specific T-cell response^47^. Indeed, CD8+ T cells are known to control viremia and viral reservoirs^48,49^. As Tregs play a role in differentiation and activation of CD8+ T cells, we speculate that in PLHIV the interaction between CD8+ T cells and Tregs is influenced by altered CCR5 dependent homing capacity of CD8+ T cells and Tregs. In the present study, the HIV reservoir was characterized by measuring CD4-associated HIV-1 DNA and RNA. Both reservoir parameters correlated positively with percentage of CCR5+ lymphocytes. Correlations between cell-associated HIV-RNA and HIV-DNA with percentages of CD4+ and CD8+ T cells were also positive, but not significant after FDR-correction. Our results are in contrast with a study that showed a negative correlation between CCR5 expression in cryopreserved PBMC and CD4+ T cells and CA HIV-1 RNA levels^50^. Methodological issues may play a role here as cryopreservation significantly declines CCR5 expression^51^, while our samples were measured immediately after blood drawing. Our results are in line with findings in subjects with CCR5-Δ32 heterozygosity that predisposes to lower CCR5 expression, both percentages and MFI^3,6^, and in whom a smaller HIV reservoir was found^6^.

Apart from viral control, CCR5 expression on immune cells is known to play a role in the development of atherosclerosis^7^, also in PLHIV^52^. Increased percentages of CCR5+ monocytes and CD8+ T cells were indeed found in asymptomatic PLHIV 6 months before a first episode of acute coronary syndrome compared to a matched control group that remained asymptomatic, suggesting an important role for CCR5 in the development of cardiovascular diseases^53^. CD8+ T cells may have both atheroprotective and atherogenic functions^54^. Here, we show increased CCR5+ percentages of all CD8+ T cell subsets. CCR5 has been linked to the accumulation of CD4+ T cells in atherosclerotic plaques^55^ and the reduction of CCR5 on Tregs may therefore compromise their immunoprotective intralesional effects^56^. Our finding of CCR5 upregulation on monocytes may also aggravate atherosclerotic plaque formation.

After exploring CCR5 expression in PLHIV and controls, we studied host factors in relation to CCR5. Higher CCR5 expression was related to male sex, older age and positive CMV serostatus in controls, as found before^9–11^, and to smoking and HIV-related factors in PLHIV. Low CD4 nadir and latest CD4 cell counts showed negative correlations with CCR5 expression, mostly on CD4+ T cells. Fast progression, low CD4 nadir and high viral loads have been associated with higher CCR5 expression in untreated PLHIV^4^. Our results indicate that these associations remain in PLHIV on suppressive cART with normalized levels of CCR5+ CD4+ T cell percentages.

Next, we assessed correlations between circulating inflammatory biomarkers and CCR5 expression. On one hand, several cytokines, such as IL-2 and IL-12, may upregulate CCR5 expression in vitro^16^. On the other hand, in subjects experimentally exposed to lipopolysaccharide, percentage of CCR5+ lymphocytes remained unchanged while the level of expression of CCR5 was upregulated^17^. Our results did not reveal significant correlations between inflammatory markers and CCR5 expression, neither in PLHIV nor controls, although a clear difference in inflammation between these two cohorts has been noticed^27^. It should be mentioned however that an acute inflammatory condition was an exclusion criterion for study participants.

Finally, associations between circulating metabolites and CCR5 expression were analyzed. No mutual metabolic pathways related to CCR5 expression could be identified in controls and PLHIV. Associations between percentages of CCR5+ CD8+ T cells and the pyruvate, propanoate and beta-Alanine metabolic pathways were found in PLHIV. The associations may rely on causal effects of energy-related pathways on CCR5 expression. CD8+ T cell metabolism is dependent on the micro-environment, including pyruvate^57^ and alanine content^58^. Moreover, it has been suggested that mitochondrial oxidative stress induces CCR5 expression^59^. Possibly the energy demands of CCR5+ CD8+ T cells are higher in PLHIV and therefore this metabolic pathway is highlighted in PLHIV only. Alternatively, increased CCR5 expression may affect energy metabolism. In this regard, our findings of associations between energy pathways and CCR5 expression on CD8+ T cells may reflect mitochondrial abnormalities caused by CCR5-dependent immune activation of CD8+ T cells. Decreased mtDNA content in CD8+ T cells of PLHIV is hypothesized to be the result of immune activation^60^, in which CCR5 plays an important role^47^. The direction of the association between energy metabolism and CCR5 expression on CD8+ T cells remains to be evaluated. Either way, our findings of associations with energy pathways might be relevant, especially since they are found for the percentage of CCR5+ CD8+ T cells, which was most altered in PLHIV.

Sphingolipid metabolism was associated with the level of CCR5 expression on several CD4+ T cells in controls. Correlation analysis with individual metabolites involved in this pathway revealed negative correlations between CCR5 MFI and several sphingolipids, including sphingosine 1-phosphate (S1P) and S1P analog dihydro-sphingosine 1-phosphate. Previous studies showed that S1P receptor 1 is highly co-expressed with CCR5 on CD4+ T cells^61^, and FTY720 (Fingolimod), a selective antagonist of S1P receptor 1, reduces the MFI of CCR5 on CD4+ T cell subsets^62^. These observations indicate that interaction of sphingolipids with their respective receptor downmodulates CCR5, which is supported by our findings. We did not see significant correlations between CCR5 MFI on different cell types and the sphingolipid pathway in PLHIV. This may be due to different sphingolipid metabolism in PLHIV. However, our study did not allow direct comparison of the metabolome between PLHIV and controls.

Our study has several limitations. First, the links with CCR5 expression rely on associations and correlations, and it remains to be evaluated if a causal relationship exists. Second, our cohort of PLHIV consisted predominantly of middle-aged men of European ancestry. Taken the differences related to genetic and non-genetic host factors that influence immune responses, our results might not be generalized to PLHIV of other sex, age, or ethnicity. Third, the distribution of demographics such as sex and age differed between PLHIV and controls. We therefore added these demographics as covariates to our models and performed an additional analysis to compare CCR5 expression in males only. PLHIV and controls also differed in medication use and presumably also in comorbidity. Fourth, CD3, CD127 and FoxP3 were not included in the flow cytometry panel. However, we may assume that CD4+ CD45RA+ CD25+ and CD4+ CD45RA-CD25++ T cell subsets represent nTreg and mTreg subsets well, as it has been shown that FoxP3 expression is proportional to CD25 expression in circulating CD4+ CD45RA+ CD25+ and CD4+ CD45RA-CD25++ cell subsets^63–65^. Fifth, we were not able to compare the concentrations of plasma markers of inflammation nor individual metabolites between the cohorts, since the measurements were performed in different batches. Sixth, CCR5Δ32 mutation is known to affect CCR5 expression and may as such affect results. However, in this study we focused on other possibly modifying factors.

In conclusion, this study shows that CCR5 expression is altered on different circulating immune cells in PLHIV on long-term suppressive cART. The higher percentage of CCR5+ CD8+ T cells in combination with lower percentages of CCR5+ Tregs in PLHIV may lead to less suppression of CD8+ T cell responses which is favorable for controlling the viral reservoir but may be less advantageous for development of non-AIDS co-morbidities. The associations between different energy pathways and percentage of CCR5+ CD8+ T cells in PLHIV, but not in controls, suggest higher energy demand of CCR5+ CD8+ T cells in PLHIV.

## Supplementary Information

Supplementary Information.

## Data Availability

The proteomics datasets from the 200HIV and 300BCG cohort are accessible from the PRIDE database (https://www.ebi.ac.uk/pride/, accession number PXD031628)^66^. Other datasets generated during and analyzed during the study are available from the corresponding author on reasonable request. R code used for the analyses is available from the author.
