# Supplementary material for: People with HIV have higher percentages of circulating CCR5+ CD8+ T cells and lower percentages of CCR5+ regulatory T cells
Source: Sci Rep. 2022 Jul 6;12:11425. doi: 10.1038/s41598-022-15646-0 (PMC9259737; doi:10.1038/s41598-022-15646-0)
Supplement: Supplementary file 1 — Supplementary Information. [file 41598_2022_15646_MOESM1_ESM.docx]

Supplemental data

**People with HIV have higher percentages of circulating CCR5+ CD8+ T cells and lower percentages of CCR5+ regulatory T cells**

**Louise E. van Eekeren^1,3*^, Vasiliki Matzaraki^1,3^, Zhenhua Zhang^4^, Lisa Wijer^1,3^, Marc Blaauw^1,3^, Marien I. de Jonge^2,3^, Linos Vandekerckhove^6^, Wim Trypsteen^6^, Leo Joosten^1,3^, Mihai G. Netea^1,3^, Quirijn de Mast^1,3^, Hans Koenen^2,3^, Yang Li^1,4,5^, André J. van der Ven^1,3^**

^1^ Department of General Internal Medicine, ^2^ Department of Laboratory Medicine, ^3^ Radboudumc Center for Infectious Diseases, ^4^ Radboud Institute for Molecular Life Sciences, Radboud University Medical Center, Nijmegen, The Netherlands

^5^ Department of Computational Biology for Individualised Medicine, Centre for Individualised Infection Medicine (CiiM) & TWINCORE, joint ventures between the Helmholtz-Centre for Infection Research (HZI) and the Hannover Medical School (MHH), Hannover, Germany

^6^ HIV Cure Research Center, Department of Internal Medicine, and Pediatrics, Ghent University & Ghent University Hospital, Belgium

*** Correspondence:**Louise E. van Eekeren
Louise.vanEekeren@radboudumc.nl

**Tables**

| Fluorochrome | FITC | ECD | APC | AF700 | APC-Cy7 | BV421 | KO |
| --- | --- | --- | --- | --- | --- | --- | --- |
| mAb | CD45RA | CD8 | CD25 | CD4 | CD195 (CCR5) | CD197 | CD45 |
| Clone | ALB11 | SFCI21Thy2D3 | 2A3 | RPA-T4 | 2D7 | G043H7 | J33 |
| Distributor | Coulter | Coulter | BD | eBioscience | BD | Biolegend | Coulter |

**Table S1** Summery of the antibody clones and the fluorochrome conjugates used for the fluorescent staining mixes. mAb = monoclonal antibody.

|  | *%* |  | *MFI* |  |
| --- | --- | --- | --- | --- |
|  | *PC 1* | *PC 2* | *PC 1* | *PC 2* |
| **CD45+ cells** | 6.94 | 8.10 | 8.23 | 1.02 |
| **Monocytes** | 2.51 | 1.63 | 5.41 | 4.88 |
| **Lymphocytes** | 10.24 | 1.43 | 9.02 | 0.87 |
| **CD4+ cells** | 8.58 | 2.36 | 8.87 | 0.63 |
| **CD4+ naive cells** | 0.83 | 6.26 | 0.60 | 35.06 |
| **CD4+ CM cells** | 4.58 | 11.34 | 6.41 | 4.49 |
| **CD4+ EM cells** | 8.87 | 5.19 | 8.62 | 0.00 |
| **CD4+ TEMRA cells** | 6.03 | 9.29e-04 | 6.31 | 0.43 |
| **CD4+ TEM cells** | 9.64 | 3.45 | 8.87 | 0.00 |
| **CD4+ nTreg** | 0.63 | 20.35 | 0.67 | 25.27 |
| **CD4+ mTreg** | 2.12 | 18.61 | 6.96 | 0.47 |
| **CD8+ cells** | 9.09 | 6.68 | 7.18 | 7.96 |
| **CD8+ naive cells** | 3.95 | 1.29 | 0.01 | 1.62 |
| **CD8+ CM cells** | 5.60 | 6.31 | 2.85 | 0.13 |
| **CD8+ EM cells** | 5.93 | 0.09 | 7.46 | 6.87 |
| **CD8+ TEMRA cells** | 7.32 | 6.79 | 4.80 | 2.65 |
| **CD8+ TEM cells** | 7.14 | 0.10 | 7.74 | 7.64 |

**Table S2** Percentage of contribution by each CCR5 expression variable to principal component 1 and 2. The parameters involved in the principal components analyses are CCR5 expression levels (expressed as percentage or MFI) of cell types that are listed in the rows. The left two columns show the results for the percentage of CCR5-expressing cells, and the right two columns show the results for the MFI of CCR5-expressing cells.

| a | *Pathway* | *Variable* | *Enrichment* | *-log(P-value)* | *Cohort* |
| --- | --- | --- | --- | --- | --- |
| **CCR5 MFI** | Sphingolipid metabolism | CD4+ cells | 8.61 | 3.24 | Healthy controls |
|  | Sphingolipid metabolism | CD4+ CM cells | 7.70 | 3.05 | Healthy controls |
|  | Sphingolipid metabolism | CD4+ TEM cells | 8.48 | 3.22 | Healthy controls |
|  | Glycerolipid metabolism | CD8+ TEMRA cells | 8.55 | 3.45 | Healthy controls |
|  | Pyrimidine metabolism | Monocytes | 3.11 | 3.11 | PLHIV |
| b | *Pathway* | *Variable* | *Enrichment* | *-log(P-value)* | *Cohort* |
| **% CCR5+ cells** | Sphingolipid metabolism | CD45+ cells | 10.84 | 3.65 | Healthy controls |
|  | Biosynthesis of unsaturated fatty acids | CD4+ cells | 3.78 | 4.72 | Healthy controls |
|  | Primary bile acid biosynthesis | CD8+ EM cells | 4.65 | 4.06 | Healthy controls |
|  | Propanoate metabolism | Lymphocytes | 4.37 | 3.96 | PLHIV |
|  | Propanoate metabolism | CD4+ cells | 6.14 | 4.99 | PLHIV |
|  | Propanoate metabolism | CD8+ cells | 4.62 | 4.13 | PLHIV |
|  | Pyruvate metabolism | CD8+ cells | 5.28 | 3.92 | PLHIV |
|  | Glycolysis / Gluconeogenesis | CD8+ cells | 3.27 | 3.01 | PLHIV |
|  | beta-Alanine metabolism | CD8+ CM cells | 3.30 | 3.12 | PLHIV |
|  | Alanine, aspartate and glutamate metabolism | CD8+ CM cells | 3.71 | 3.05 | PLHIV |
|  | beta-Alanine metabolism | CD8+ TEMRA cells | 4.29 | 3.97 | PLHIV |
|  | beta-Alanine metabolism | CD8+ TEM cells | 5.30 | 3.34 | PLHIV |

**Table S3 a and b** Metabolic pathways correlated to CCR5 expression (a: MFI, b: %) in PLHIV and healthy controls. This table depicts pathways with an unadjusted P < 0.001. Enrichment factor refers to the ratio between the number of significant pathway hits and the expected number of compound hits within the pathway.

**Figures**

| 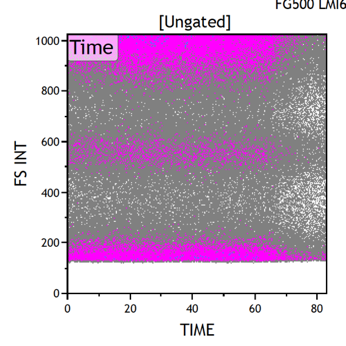 | 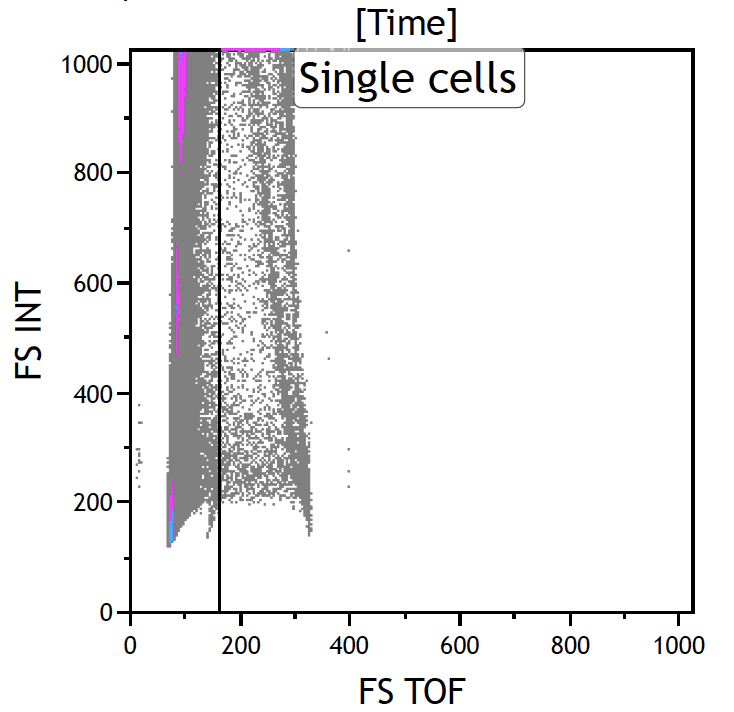 | 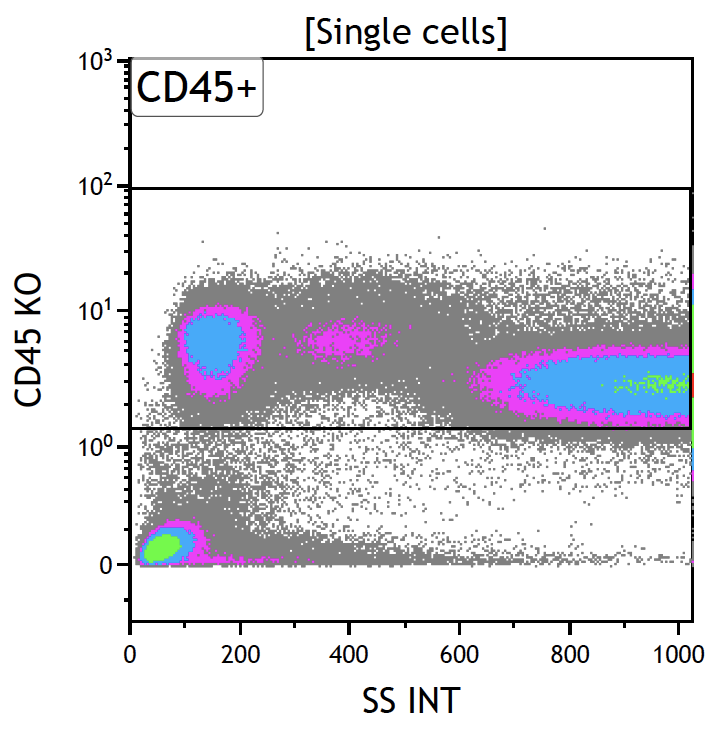 | 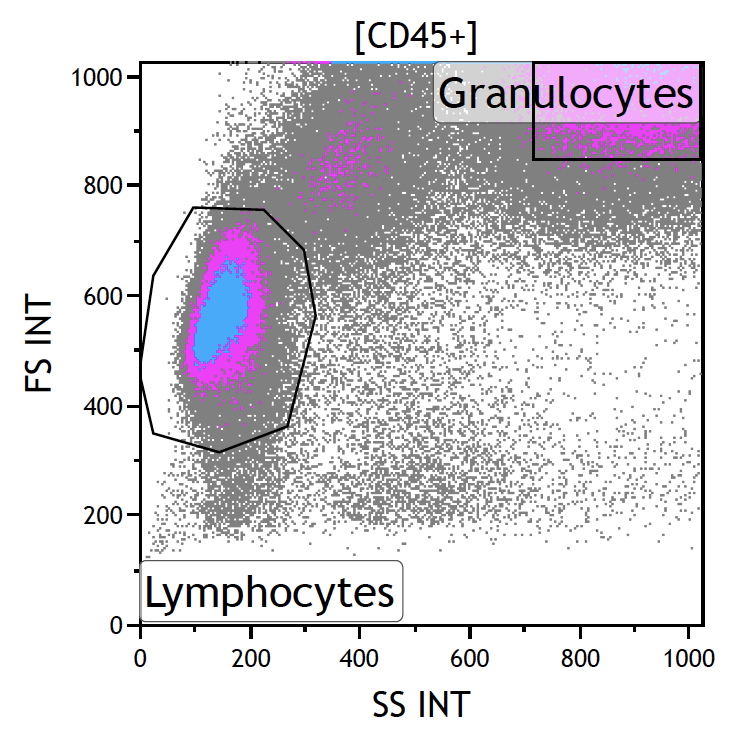 |
| --- | --- | --- | --- |
| 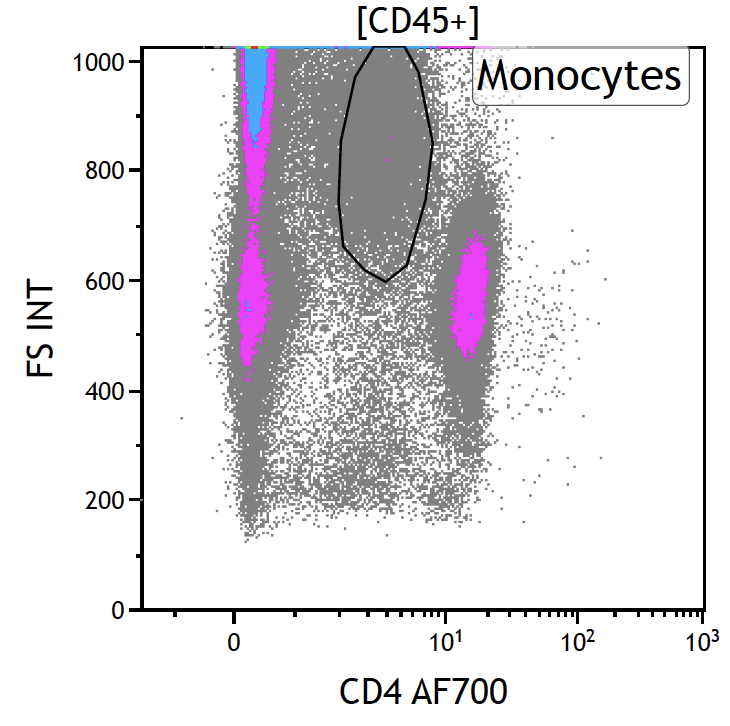 | 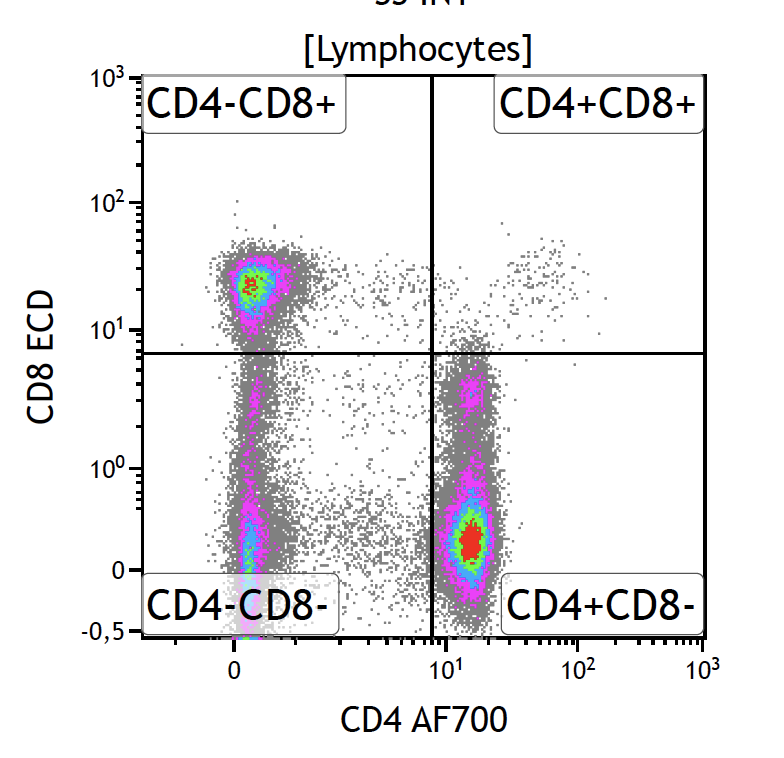 | 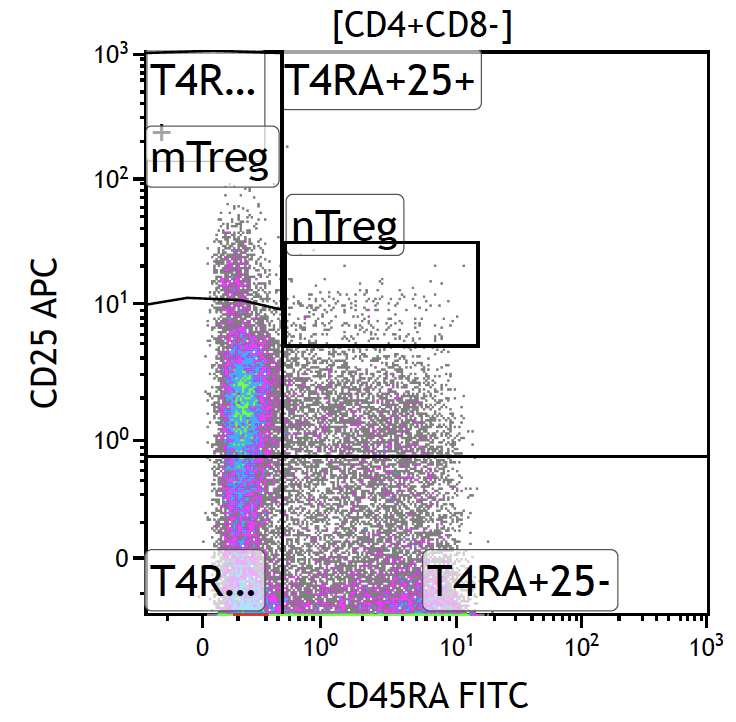 | 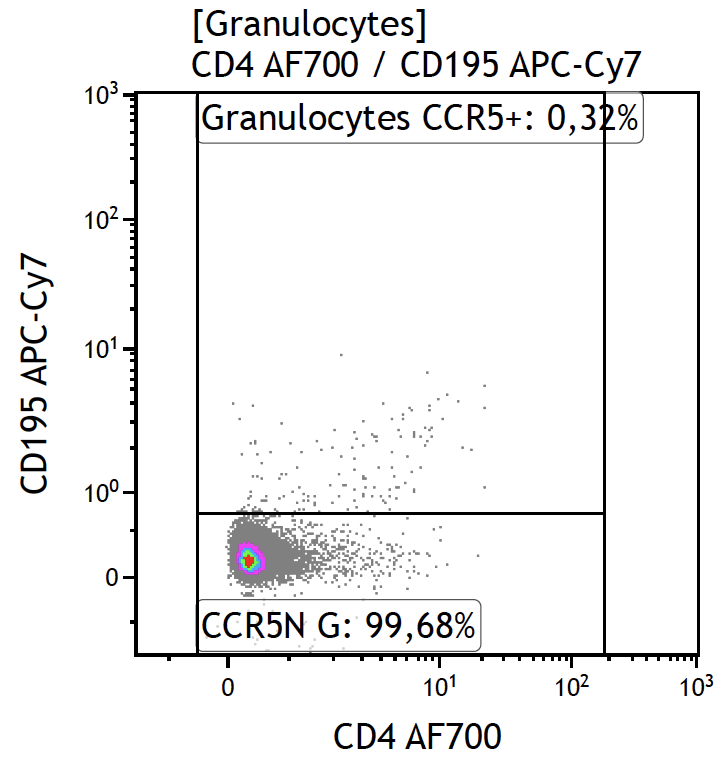 |
| 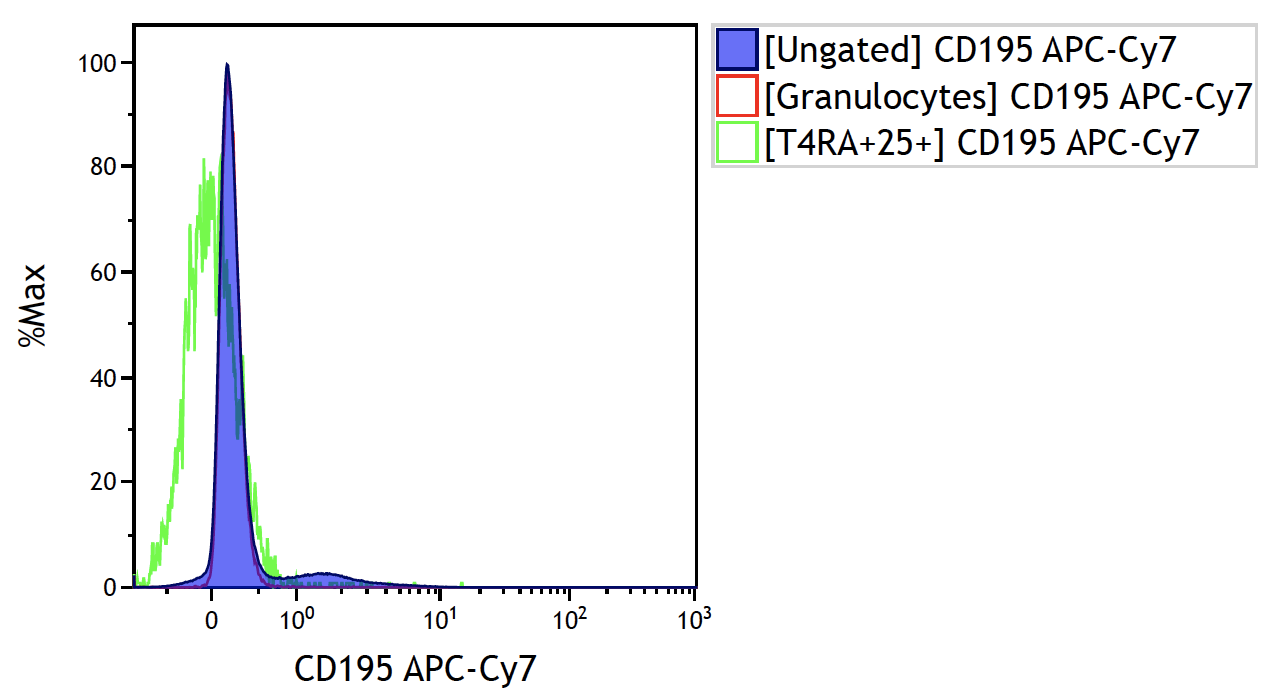 | | 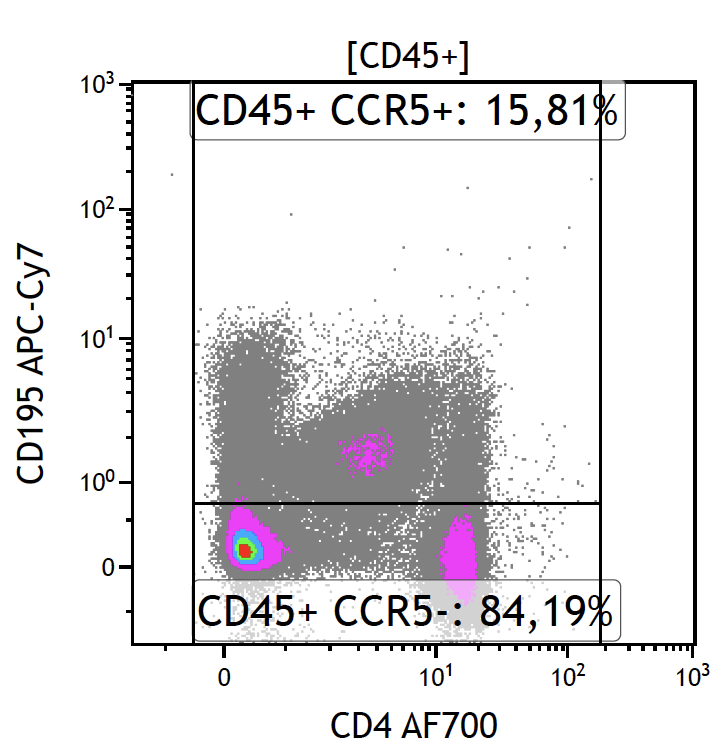 | 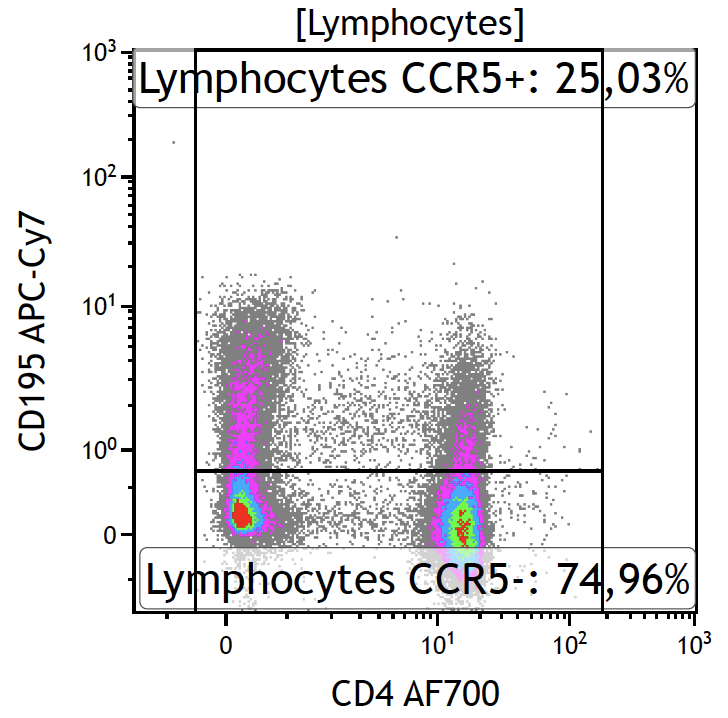 |
| 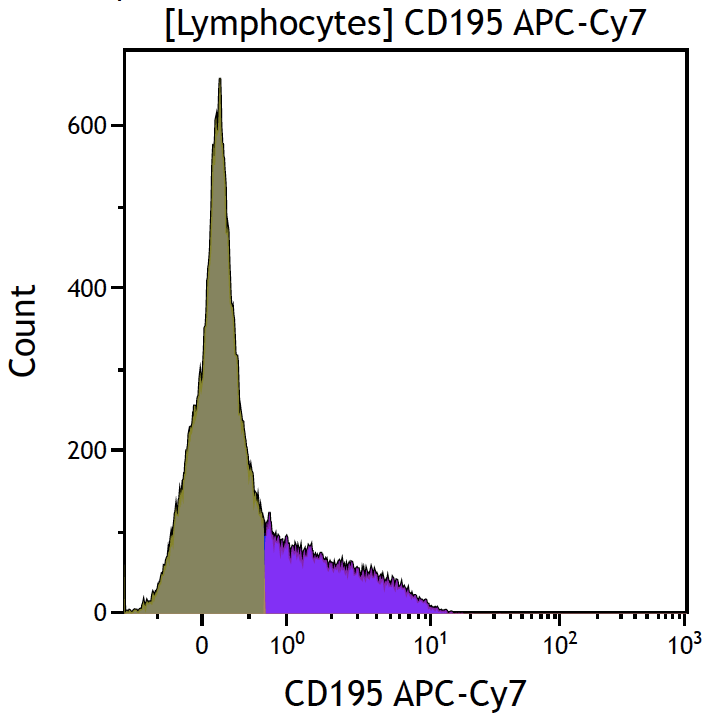 | 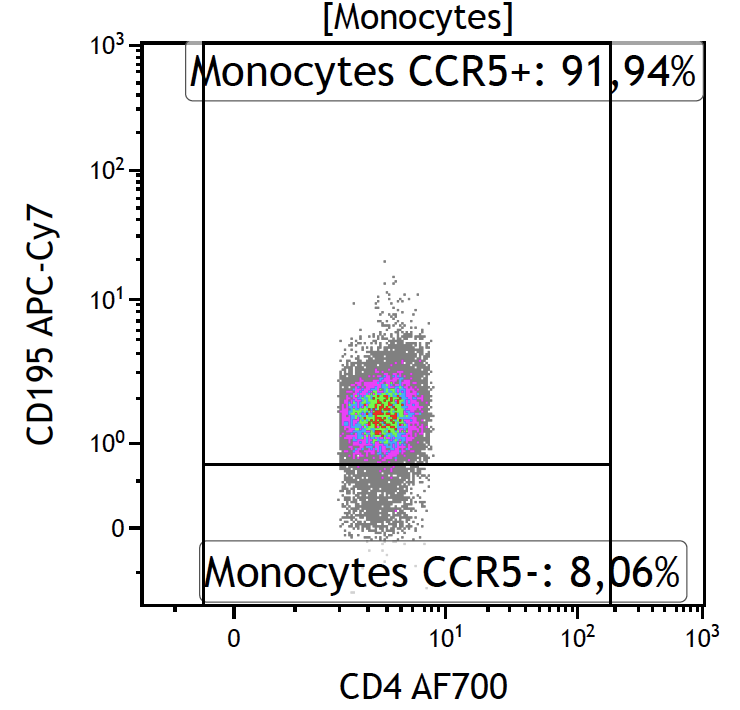 | 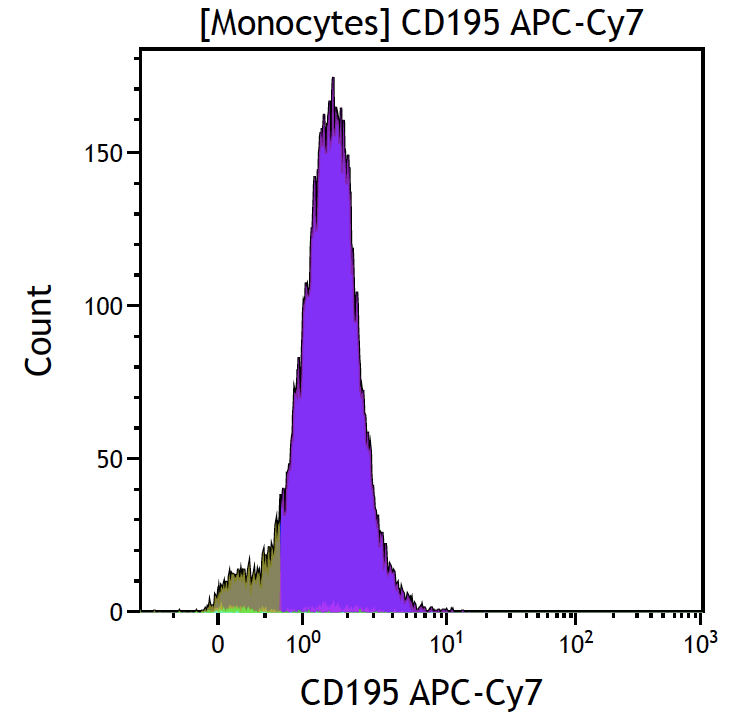 | 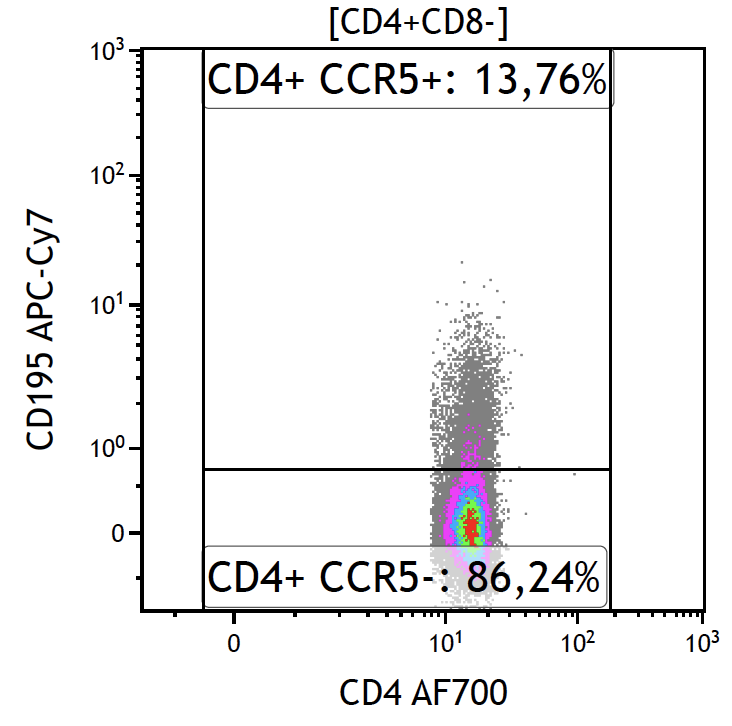 |
| 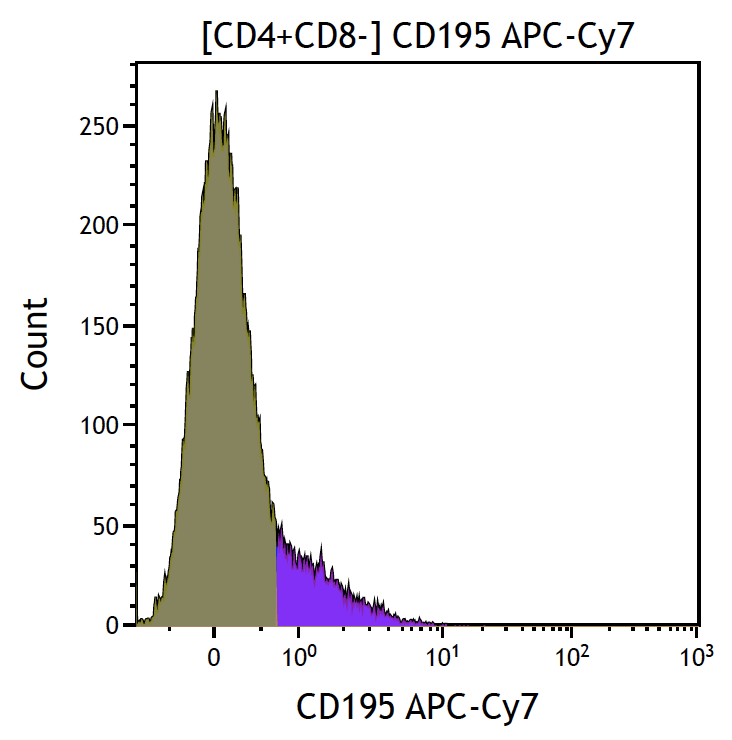 | 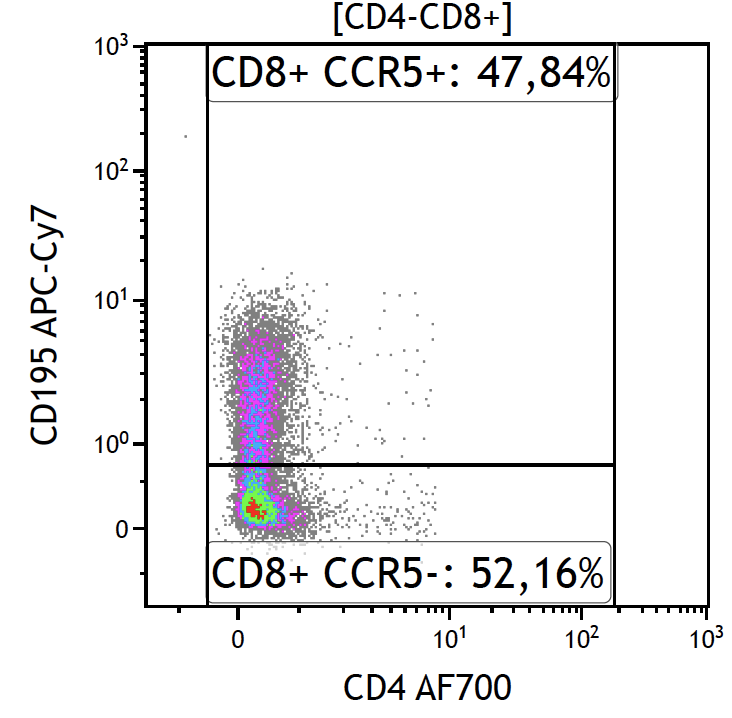 | 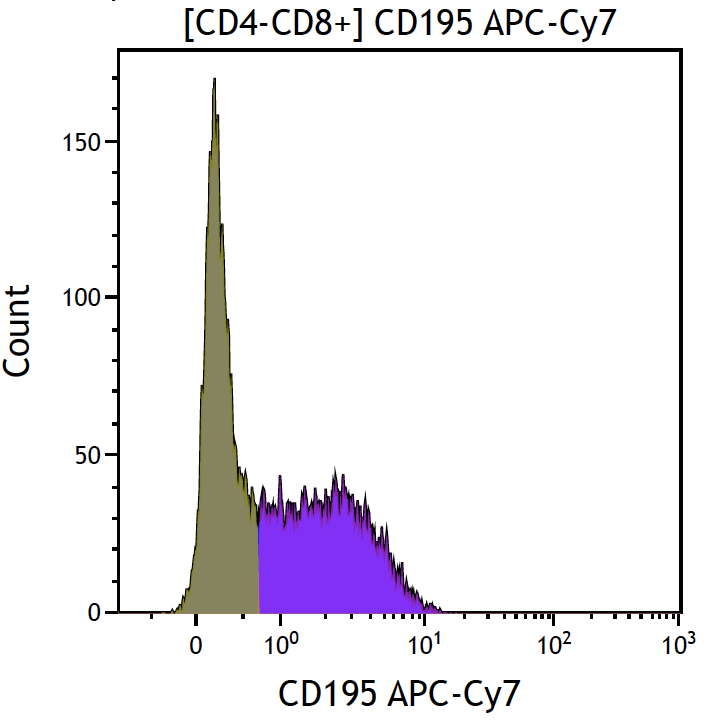 | 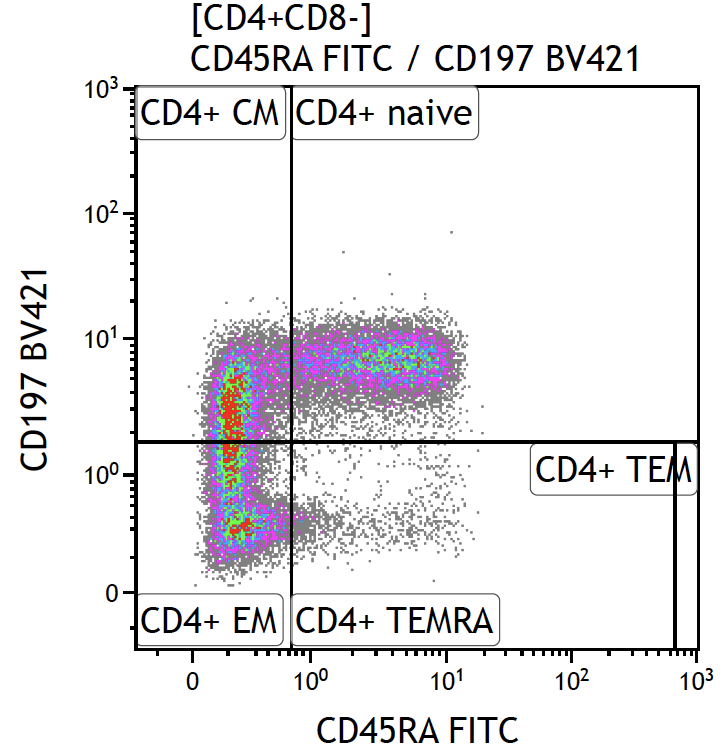 |
| 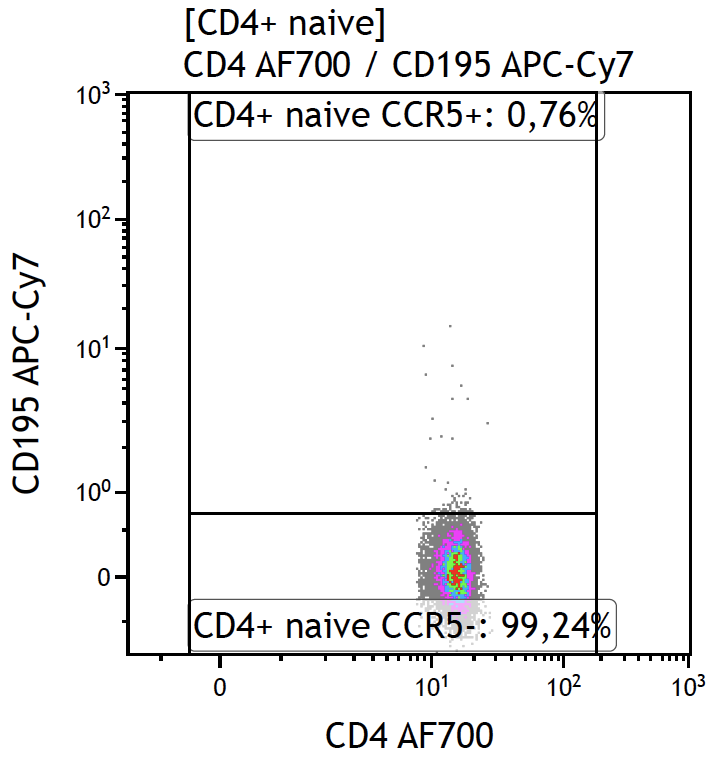 | 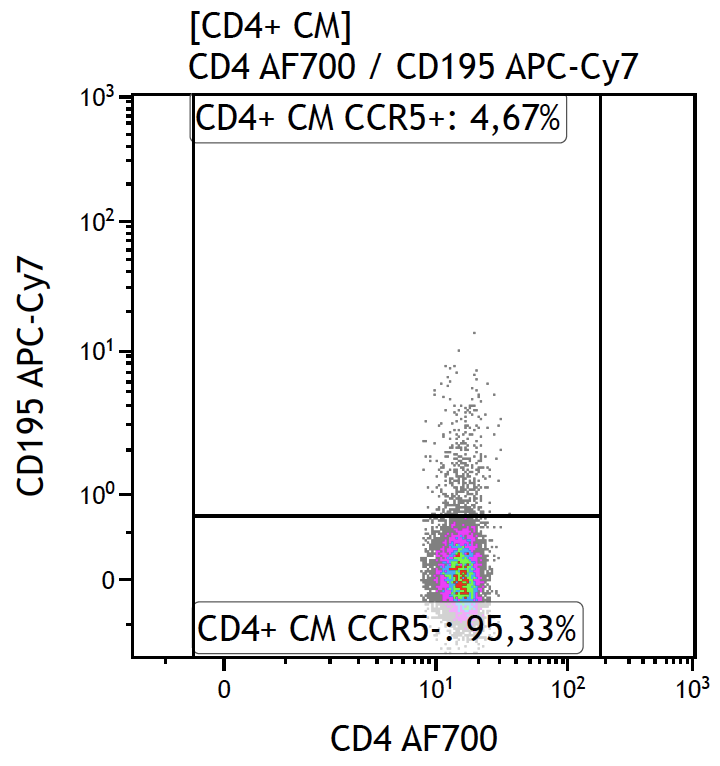 | 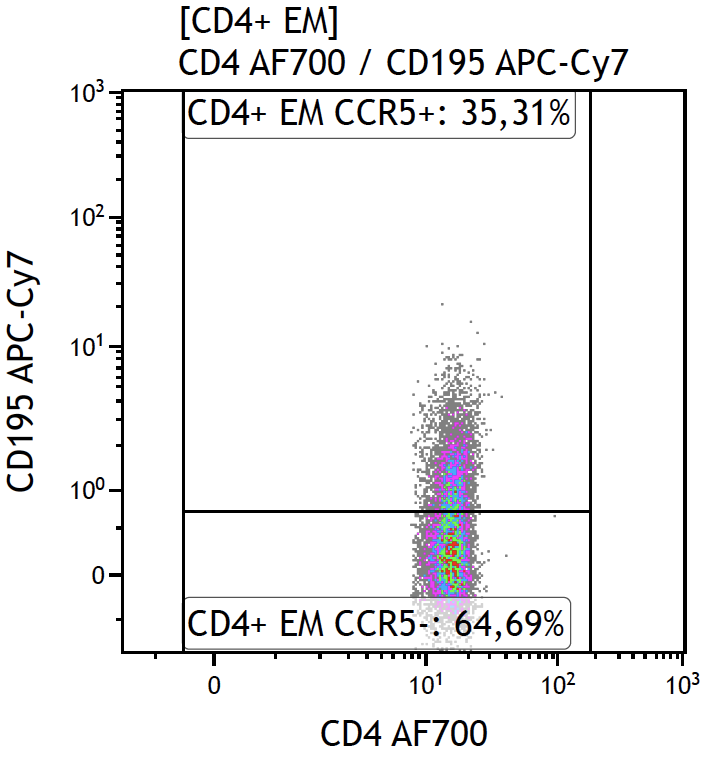 | 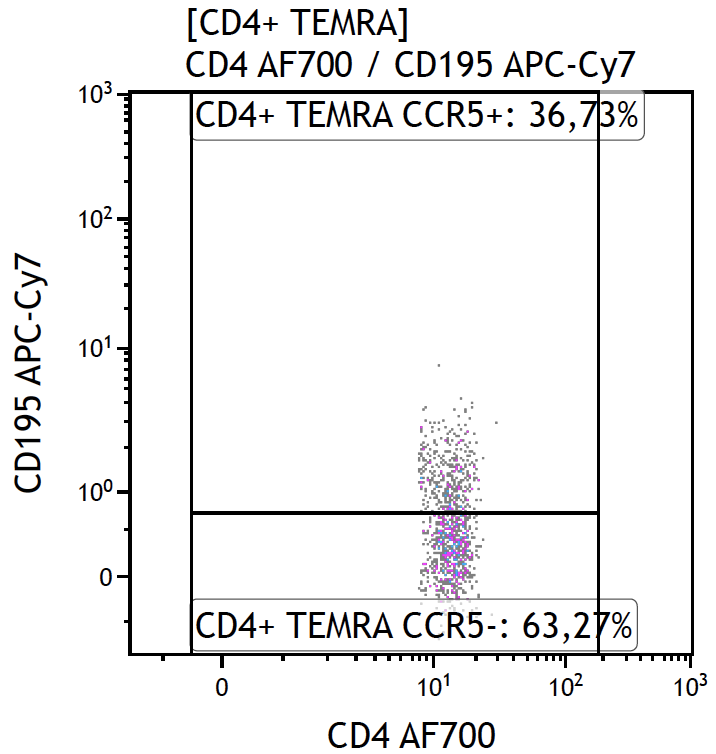 |
| 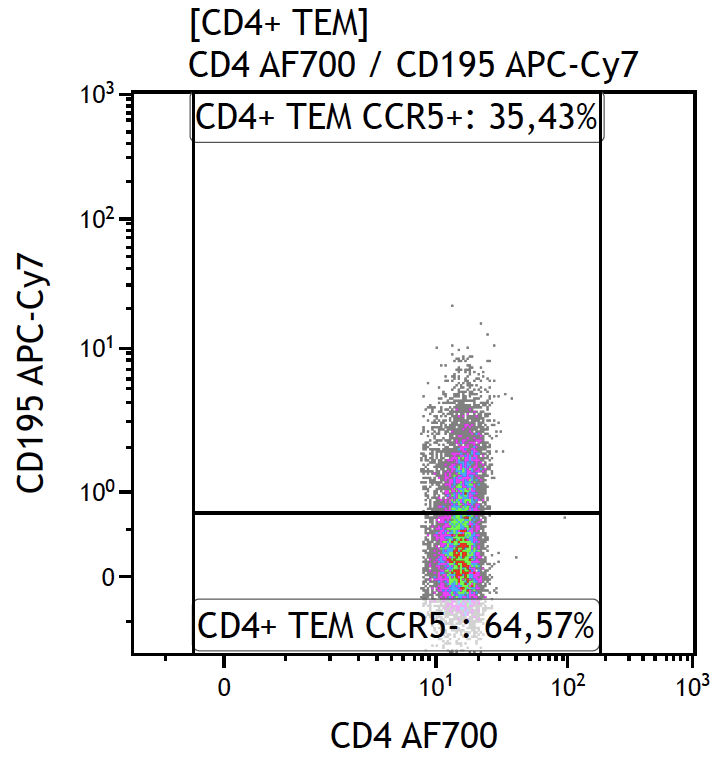 | 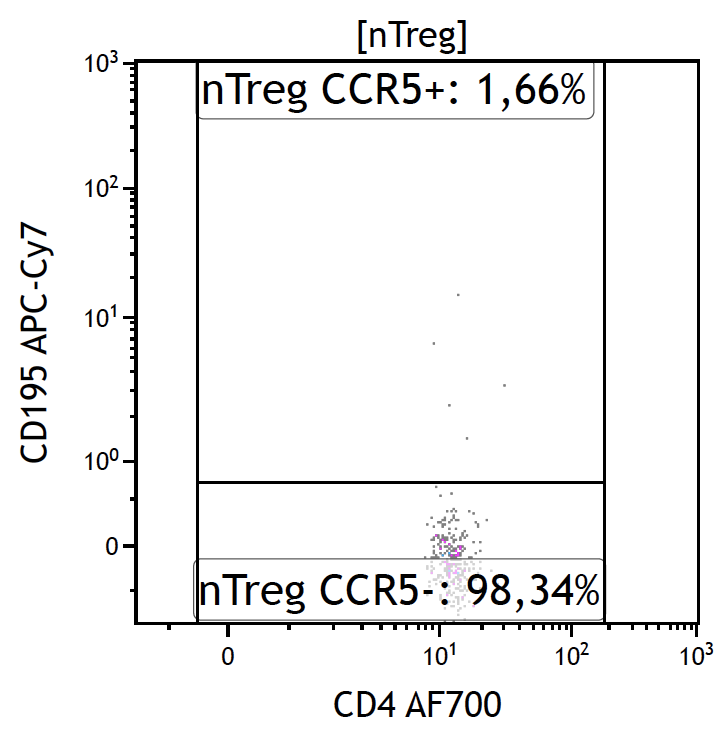 | 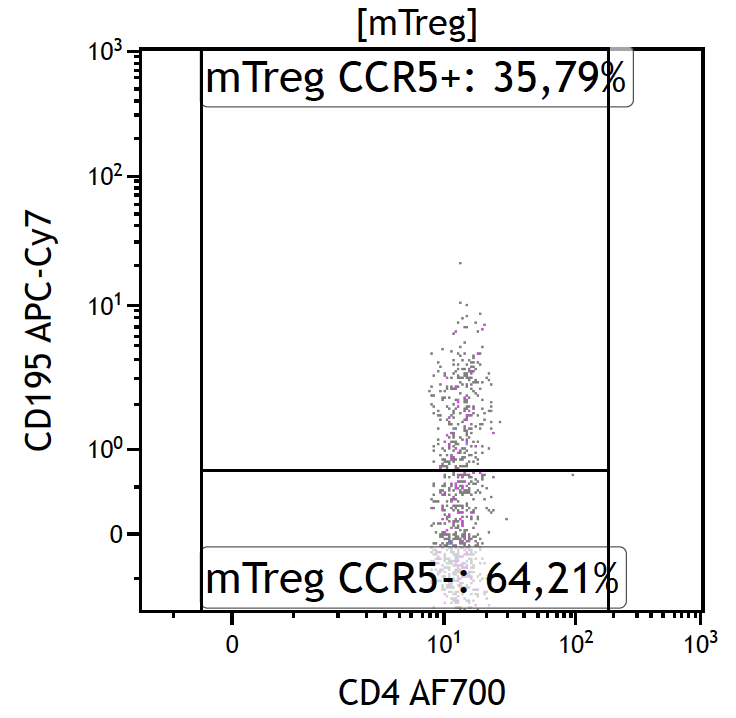 | 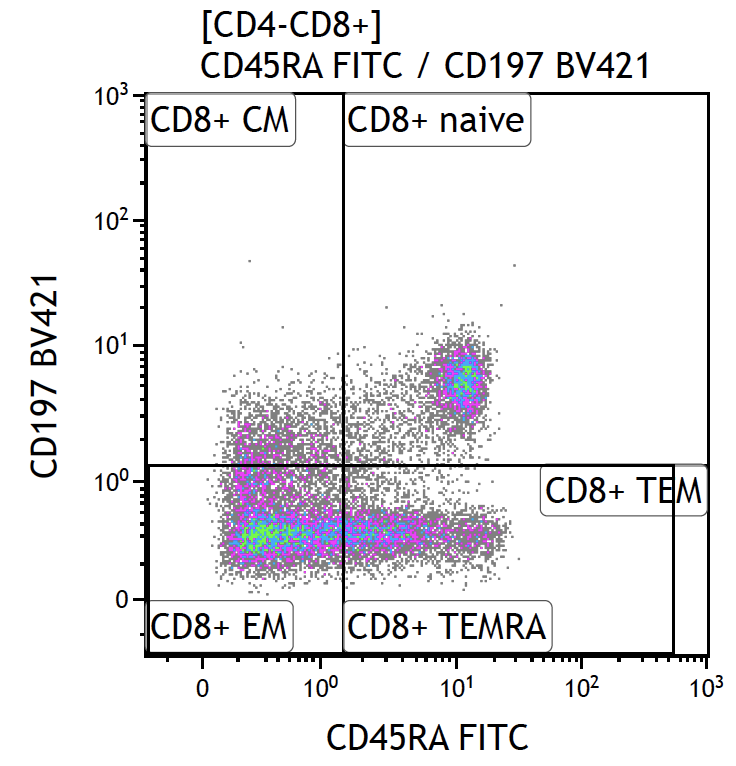 |
| 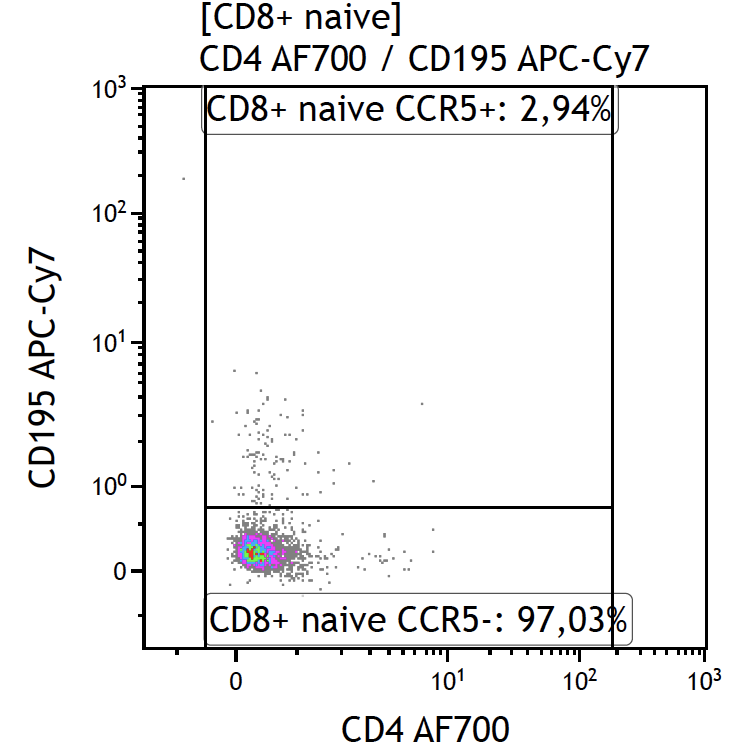 | 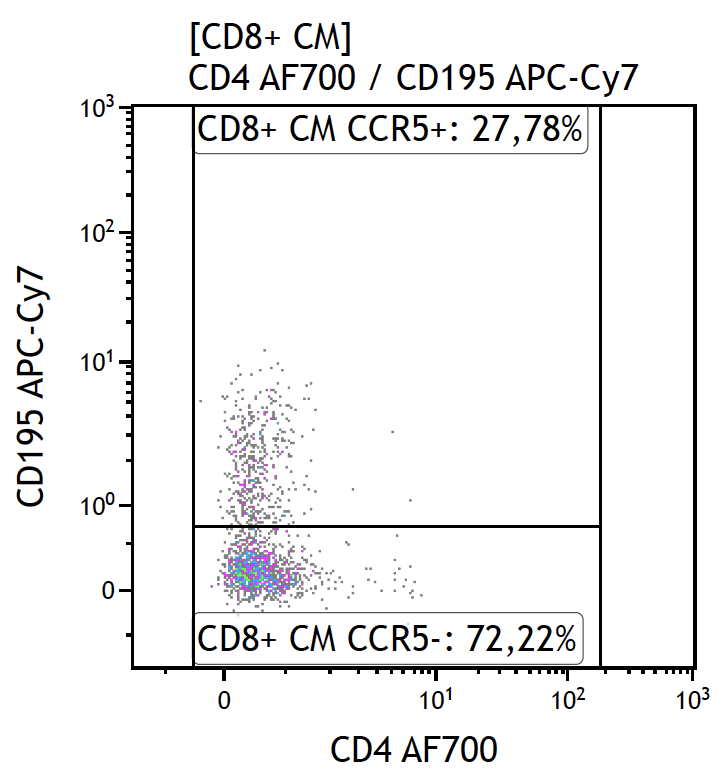 | 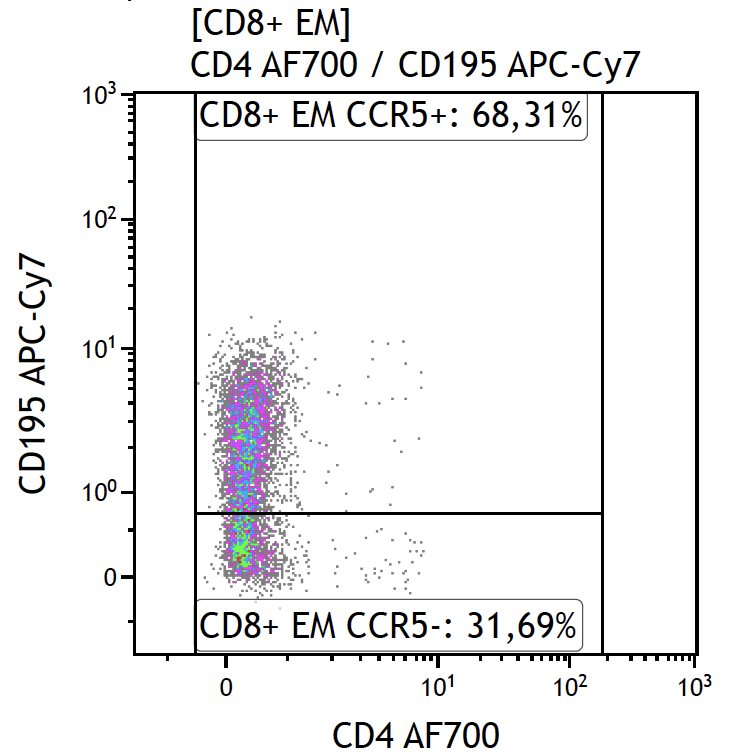 | 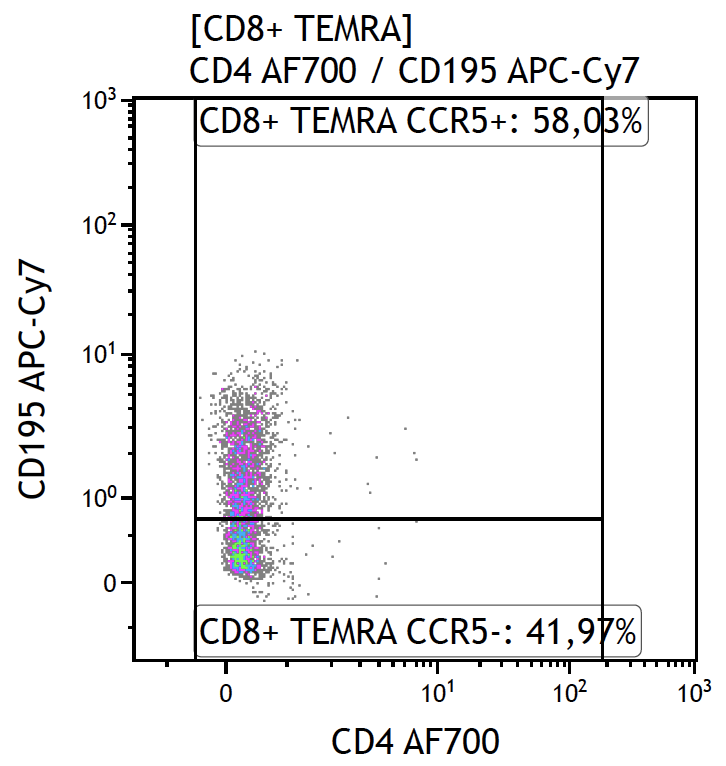 |
| 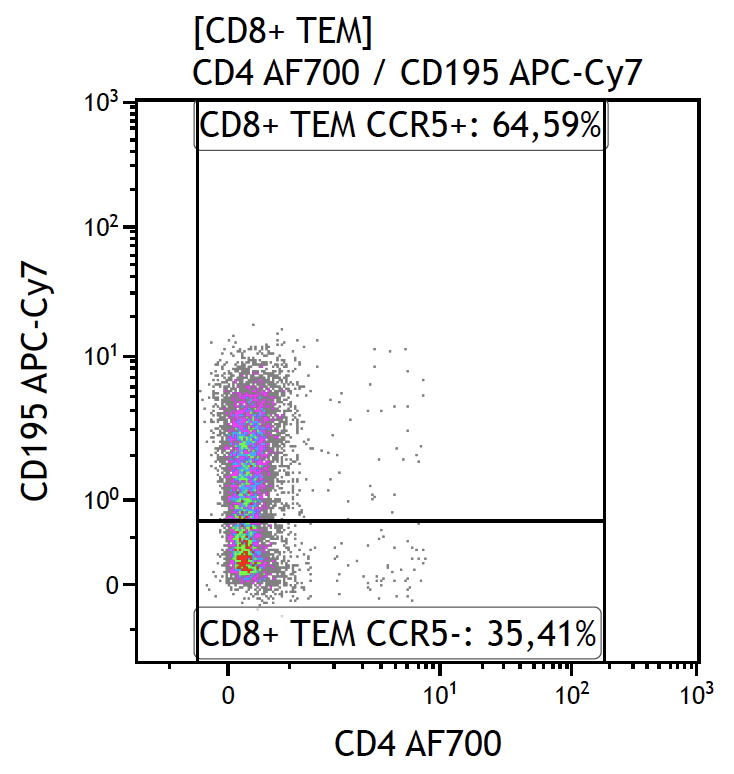 |  |  |  |

**Figure S1** Example of the gating strategy. CD45+ cells were identified by gating on live and single cells and subsequently on CD45+ cells. Within the CD45+ cells, lymphocytes and monocytes were identified by granularity (side scatter) and size (forward scatter). Lymphocytes were further classified into different subsets of CD4+(CD8-) T cells and (CD4-) CD8+ T cells.

**Figure S2** Boxplots showing the percentage of various CCR5+ cell subsets from people living with HIV (PLHIV, green colour) versus healthy controls (orange colour). Comparison between the two groups was performed by Mann-Whitney U test. Abbreviations: CM T cells = central memory T cells, EM T cells = effector memory T cells, TEMRA cells = T effector memory cells expressing CD45RA, nTreg = naïve regulatory T cells, mTreg = memory regulatory T cells.

**Figure S3** Boxplots showing the levels of CCR5 MFI on different cell subsets in people living with HIV (PLHIV, green colour) and healthy controls (orange colour). Comparison between the groups is performed by Mann-Whitney U test. Abbreviations: CM T cells = central memory T cells, EM T cells = effector memory T cells, TEMRA cells = T effector memory cells expressing CD45RA, nTreg = naïve regulatory T cells, mTreg = memory regulatory T cells.


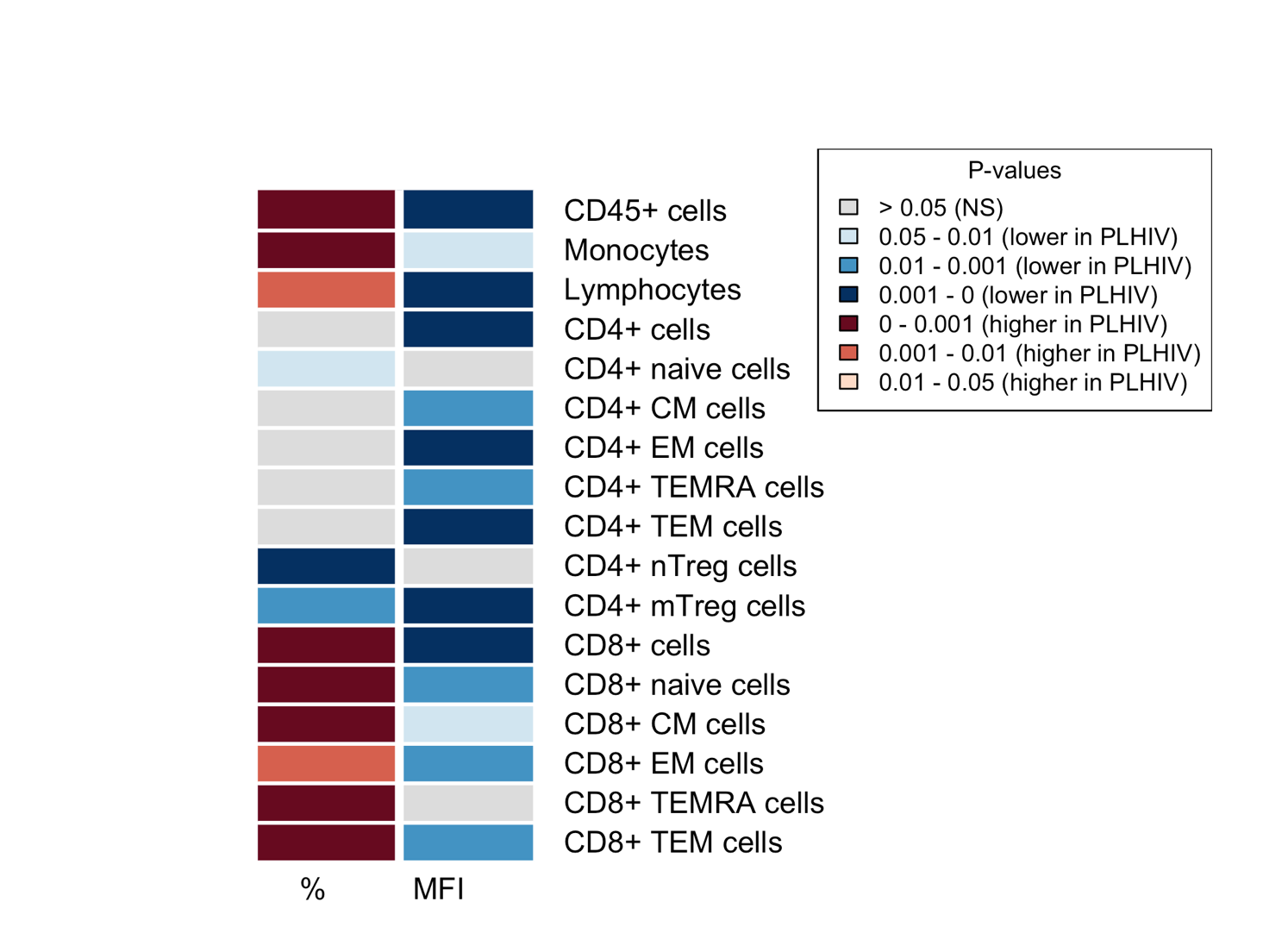


**Figure S4** CCR5 expression in circulating immune cells in male PLHIV versus male controls. The left column shows differences in the percentage (%) of CCR5+ cells, the right column shows differences in the CCR5 mean fluorescence intensity (MFI). Significance (FDR corrected) of the difference in CCR5 expression between PLHIV and controls is presented. Multiple linear regression analysis was used with age, sex, CMV-serostatus and smoking as covariables. Blue colour refers to lower CCR5 expression in PLHIV, while red refers to higher expression in PLHIV compared to controls.

**Figure S5** Results from multiple linear regression analysis between host factors and CCR5 expression (% and MFI) on immune cell subsets while adjusting for HIV-specific variables. The colour indicates the direction of association (blue for negative association, red for positive association), whereas the brightness of the colour represents the level of significance. Unadjusted p-values are presented. NS = not significant.

**b**

**a**

**Figure S6** Correlation plots of circulating inflammatory proteins and CCR5 expression (% and MFI) on immune cell subsets in (a) healthy controls and (b) people living with HIV (PLHIV). Partial Spearman’s rank correlation was performed with age and sex as covariables. The colours represent the strength of the Spearman’s rank correlations coefficients. Unadjusted P-values are presented and P-values that were not statistically significant (P > 0.05) are represented as blank squares. The inflammatory proteins are ordered from the lowest to the highest p-value. The lowest P-value was found for the correlation between CCL4 and percentage of CCR5+ CD8+ cells (P = 1 x 10^-4^)) in PLHIV.

**Figure S7** Correlation plot between metabolic compounds involved in propanoate metabolism and percentages of CCR5+ cells in PLHIV. The y-axis shows the measured IonMz values combined with their corresponding KEGG ID according to the mummichog algorithm. Strength and direction of the correlation coefficients are only shown for P-values < 0.05 (not FDR-corrected). Spearman’s rank correlation was used with age, sex, and CMV serostatus as covariables.

**Figure S8** Correlation plot between metabolic compounds involved in pyruvate metabolism and percentage of CCR5+ cells in PLHIV. The y-axis shows the measured IonMz values combined with their corresponding KEGG ID according to the mummichog algorithm. Strength and direction of the correlation coefficients are only shown for P-values < 0.05 (not FDR-corrected). Spearman’s rank correlation was used with age, sex, and CMV serostatus as covariables.

**Figure S9** Correlation plot between metabolic compounds involved in beta-Alanine metabolism and percentage of CCR5+ cells in PLHIV. The y-axis shows the measured IonMz values combined with their corresponding KEGG ID according to the mummichog algorithm. Strength and direction of the correlation coefficients are only shown for P-values < 0.05 (not FDR-corrected). Spearman’s rank correlation was used with age, sex, and CMV serostatus as covariables.

**Figure S10** Correlation plot between metabolic compounds involved in sphingolipid metabolism and CCR5 MFI in healthy controls. The y-axis shows the measured IonMz values combined with their corresponding KEGG ID according to the mummichog algorithm. Strength and direction of the correlation coefficients are only shown for P-values < 0.05 (not FDR-corrected). Spearman’s rank correlation was used with age, sex, and CMV serostatus as covariables.
